# Atlas of phosphoinositide signatures in the retina identifies heterogeneity between cell types

**DOI:** 10.1093/pnasnexus/pgad063

**Published:** 2023-03-03

**Authors:** Ammaji Rajala, Rahul Rajala, Gopa Kumar Gopinadhan Nair, Raju V S Rajala

**Affiliations:** Department of Ophthalmology, University of Oklahoma Health Sciences Center, 608 Stanton L. Young Blvd, Oklahoma City, OK 73104, USA; Dean McGee Eye Institute, 608 Stanton L. Young Blvd, Oklahoma City, OK 73104, USA; Department of Cell Biology, University of Oklahoma Health Sciences Center, 608 Stanton L. Young Blvd, Oklahoma City, OK 73104, USA; Cardiovascular Biology Program, Oklahoma Medical Research Foundation, 825 NE 13th St, Oklahoma City, OK 73104, USA; Department of Ophthalmology, University of Oklahoma Health Sciences Center, 608 Stanton L. Young Blvd, Oklahoma City, OK 73104, USA; Dean McGee Eye Institute, 608 Stanton L. Young Blvd, Oklahoma City, OK 73104, USA; Department of Ophthalmology, University of Oklahoma Health Sciences Center, 608 Stanton L. Young Blvd, Oklahoma City, OK 73104, USA; Dean McGee Eye Institute, 608 Stanton L. Young Blvd, Oklahoma City, OK 73104, USA; Department of Cell Biology, University of Oklahoma Health Sciences Center, 608 Stanton L. Young Blvd, Oklahoma City, OK 73104, USA; Department of Physiology, University of Oklahoma Health Sciences Center, 608 Stanton L. Young Blvd, Oklahoma City, OK 73104, USA

**Keywords:** phosphoinositides, phosphoinositide kinases, phosphoinositide phosphatases, retina, retinal diseases

## Abstract

Phosphoinositides (PIPs) are a family of minor acidic phospholipids in the cell membrane. Phosphoinositide (PI) kinases and phosphatases can rapidly convert one PIP product into another resulting in the generation of seven distinct PIPs. The retina is a heterogeneous tissue composed of several cell types. In the mammalian genome, around 50 genes encode PI kinases and PI phosphatases; however, there are no studies describing the distribution of these enzymes in the various retinal cell types. Using translating ribosome affinity purification, we have identified the in vivo distribution of PI-converting enzymes from the rod, cone, retinal pigment epithelium (RPE), Müller glia, and retinal ganglion cells, generating a physiological atlas for PI-converting enzyme expression in the retina. The retinal neurons, rods, cones, and RGCs, are characterized by the enrichment of PI-converting enzymes, whereas the Müller glia and RPE are characterized by the depletion of these enzymes. We also found distinct differences between the expression of PI kinases and PI phosphatases in each retinal cell type. Since mutations in PI-converting enzymes are linked to human diseases including retinal diseases, the results of this study will provide a guide for what cell types are likely to be affected by retinal degenerative diseases brought on by changes in PI metabolism.

**Significance Statement**

Mutations in PI-converting enzymes result in retinal degeneration. In the mammalian genome, around 50 genes encode phosphoinositide (PI) kinases and PI phosphatases that generate seven distinct phosphoinositides. Here, we investigate which PI kinases and PI phosphatases are expressed in different retinal cells. We isolated rod, cone, RPE, Müller, and retinal ganglion cell-specific actively translating messenger RNAs and identified the heterogeneous expression of PI kinases and PI phosphatases. We found that rods, cones, and RGCs show greater enrichment of PI kinases and PI phosphatases, whereas depletion of these enzymes in Müller glia and RPE. The results reported in this study provide a physiological atlas identifying what retinal cells are likely to detrimentally respond to changes in PI metabolism.

## Introduction

Phosphoinositides are low-abundant but critical, cellular membrane phospholipids (1–5). Reversible phosphorylation of phosphatidylinositol (PI) at the free-hydroxyl groups results in the generation of seven distinct phosphorylated second messenger molecules commonly known as phosphatidylinositol phosphatases or phosphoinositides or PIPs (1). The PIPs are dynamically converted from one form to another through the action of PI kinases and PI phosphatases (1–4). PIPs are widespread signaling molecules that associate with membrane and cytosolic proteins containing phospholipid-binding domains that directly bind PIPs and are tethered to the cell membrane (6, 7). PIPs regulate a wide range of cellular functions, including membrane budding and fusion, cytoskeletal assembly, vesicular transport, ciliogenesis, and signal transduction (1–5, 7).

In the nervous system, PIPs play an important role in neural function (8); they regulate neuroexocytosis (9) and coordinate the cascade of synaptic events leading to neurotransmission (10). With the advent of sequencing the human genome, mutations in PI metabolizing enzymes and components of the PIP signaling pathway have been linked to various human diseases (8, 11). Monogenic brain disorders are reported as a loss of function or mutation in genes of the PIP signaling pathway (8, 11). Furthermore, genome-wide association (GWAS) studies identified strong single-nucleotide polymorphism-risk alleles (SNP) in PIP signaling genes to brain disorders (8). The retina is an extension of the brain formed embryonically from neural tissues and connected to the brain proper by the optic nerve (12); it is a complex heterogeneous tissue consisting of several layers of neurons, interconnected by synapses, and supported by an outer layer of pigmented epithelial cells (13). The neurons of the retina constitute rod photoreceptor cells, cone photoreceptor cells, bipolar cells, Müller cells, amacrine cells, horizontal cells, and ganglion cells (13). There is a paucity of studies available on the role of PIPs in the retina (2, 3). However, altered PI metabolism brought on by altered activities of PI-converting enzymes leads to altered retinal function, e.g. mutations in the PI phosphatase OCRL are associated with severe ciliopathy called oculocerebrorenal syndrome of Lowe (14, 15). All OCRL patients develop dense bilateral congenital cataracts and 50% of patients develop congenital glaucoma (16). Increased intraocular pressure (IOP) is associated with the development and progression of glaucoma (17). It has been shown that optogenetic stimulation of OCRL, an inositol 5-phosphatase, increases aqueous humor and lower IOP in vivo (17, 18). PI phosphatase INPP5E is an inherited retinal degeneration gene that regulates the cilia transition zone and mutations cause Joubert syndrome (19). In addition, mutations in genes encoding PIP-binding proteins are frequently associated with inherited retinal degeneration. Mutations in TULP cause retinitis pigmentosa (20), mutations in BBS genes cause Bardet-Biedl syndrome and retinitis pigmentosa (21), and mutations in phosphatidyl inositol transfer protein PIT3NM3 are associated with autosomal dominant cone-rod dystrophy CORD5 (22). The serine/threonine kinase AKT3 binds to D3-PIPs and GWAS study has identified two AKT3 SNP-risk alleles associated with diabetic retinopathy (8). Other GWAS studies have identified the FERMT2 SNP-risk allele associated with glaucoma, FERMT2 is a D3-PIP-binding protein (8).

The progress in the retina research on PIPs is rather slow due to the lack of information on the expression of various PI kinases and PI phosphatases and other PIP regulatory proteins in the retina. In the mammalian genome, more than 50 genes encode PI kinases and PI phosphatases (23). Furthermore, we lack knowledge of the retina cell-type-specific regulation of PI metabolism. Altered PI metabolism is implicated in various retinal pathologies (2, 3); thus, it is essential to identify cell-specific expression of PI-converting enzymes in normal physiology. In this study, we have employed an in vivo Translating Ribosome Affinity Purification (TRAP) model coupled with retina cell-specific Cre-drivers to isolate actively translating mRNAs of PI kinases, and PI phosphatases from the rod, cone, Müller cells, RPE, and retinal ganglion cells (RGCs) to generate an atlas of their cell-type-specific expression of PI-converting enzymes. These genes were read using qRT-PCR, as we could hone in and target our analysis to various genes, a task not possible with sequencing-based approaches, as there is a stochastic chance sequencing does not get a read on a particular gene.

## Results

### Characterization of PI-converting enzymes in isolated photoreceptors

**Fig. 1.**
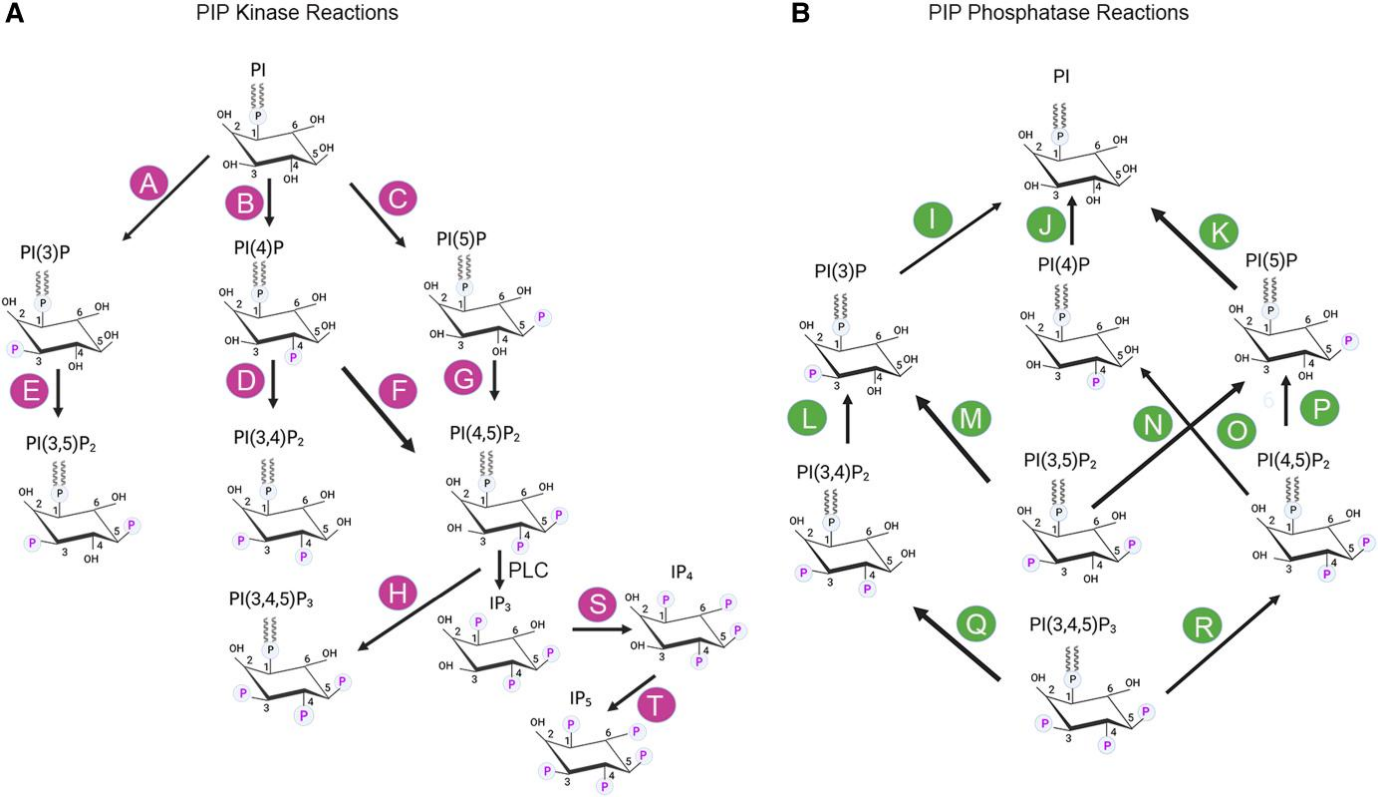
Generation of seven phosphoinositides by the action of PI kinases and PI phosphatases. Kinase reactions are shown in panel (A). Phosphatase reactions are shown in Panel (B). Each kinase and phosphatase reaction is labeled with alphabets (A–T). Kinase reactions: A, D, H—class I PI3K; A, D—class II PI3K; A—class III PI3K; B- PI4K IIα, PI4K IIβ, PI4K IIIα, PI4K IIIβ; F— PIPK 1α, PIPK Iβ, PIPK Iγ; G— PIPK IIα, PIPK IIβ, PIPK IIγ; C, E— PIPK III (PIKFyve). Phosphatase reactions: I, R— PTEN; I, N—MTM1; MTMR1, MTMR2, MTMR3, MTMR4, MTMR6, MTMR7, MTMR14, I—MTMR8; L— INPP4A, INPP4B; P—TMEM55A, TMEM55B; I, J, M, O, P, Q—SYNJ1, SYNJ2; O, Q—OCRL1, SKIP; O, Q—INPP5B; M, O, Q—INPP5E; K—PTPMT1; I, J, M—SACMIL; M—FIG4; H, S, T—IPMK. This figure was created with BioRender.com.

**Fig. 2.**
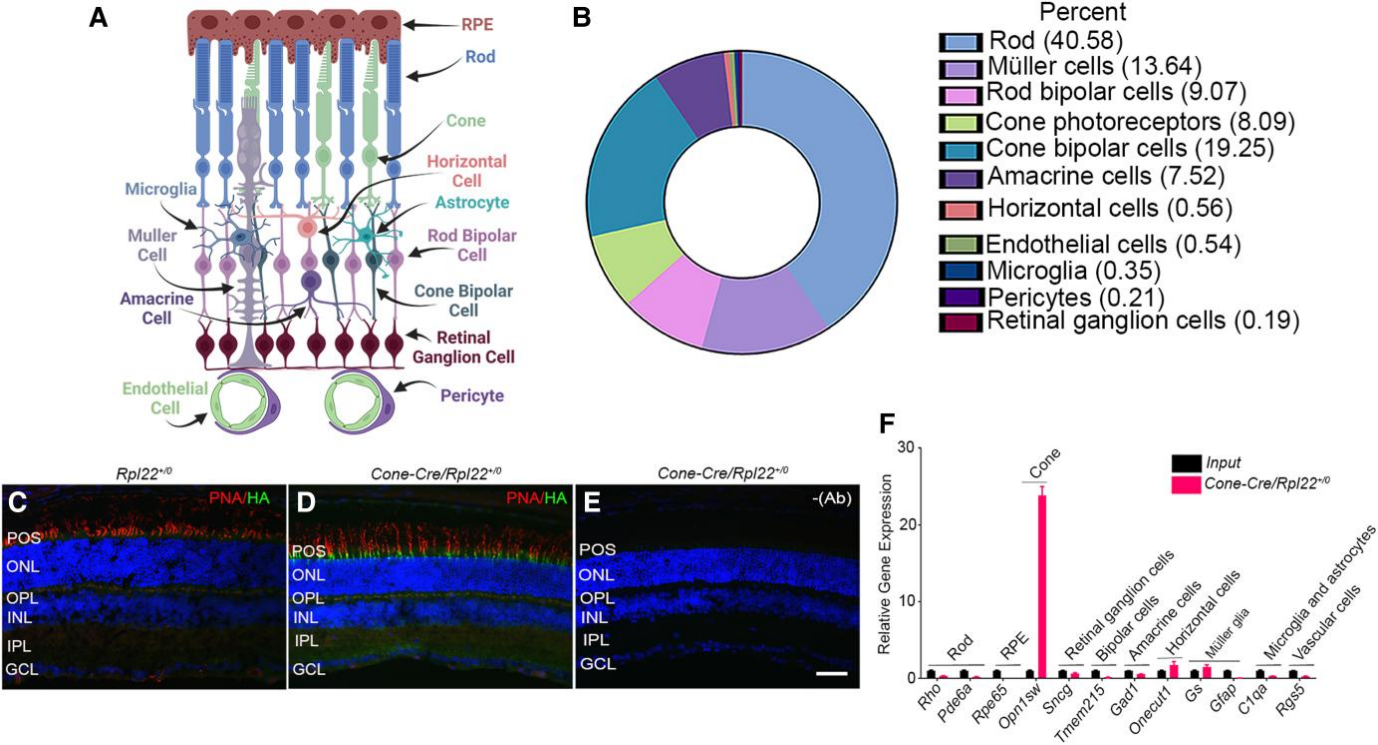
Retina structure, cell types, and characterization of Cone-Cre/Rpl22 mouse line. Cross-section of the retina (A) and percentage of retinal cells (B). The pie chart was generated from a previously published paper (25). Retinal sections from *Rpl22*^+/0^ (C) and *Cone-Cre/Rpl22*^+/0^ (D) mice were stained with anti-HA antibodies. Omission of HA antibody (E). Equal amounts of mRNA from the retina (input) and cone-Cre IP samples were used to measure retinal cell markers by qPCR, and the data normalized to *Rpl37* and *Rpl38* levels (F). Data mean ± *SEM* (*n* = *3*). Panel (A) was created with BioRender.com.

The parent molecule phosphatidylinositol is phosphorylated by PI-3, PI-4, and PI-5 kinases, producing seven distinct phosphoinositides; phosphoinositide levels in cells are dynamically regulated through dephosphorylation by PI-3, PI-4, and PI-5 phosphatases (Fig. 1, Tables 1 and 2). In general, multiple kinases and multiple phosphatases can use the same substrate to generate other PIPs (Fig. 1, Tables 1 and 2). The retina is a complex tissue composed of several cell types (Fig. 2A) with different percentages in mouse retina (Fig. 2B). Intact photoreceptors (PR) can be isolated with Opti-prep gradient centrifugation (24); however, gene expression has never been analyzed. Intact PR were isolated on Opti-prep (Figure S1A) and are reactive towards rod photoreceptor marker, rhodopsin, and cone-photoreceptor marker, M-opsin antibodies (Figure S1B). These observations suggest the presence of both rod and cone photoreceptors (Figure S1B, C). qRT-PCR analysis of RNA isolated from PR with various retinal-cell-specific primers (Table S1) shows increased enrichment of rod, and cone markers in PR compared to input which represents total retina (Figure S1D). However, markers of amacrine cells, microglia, astrocytes, and vascular cells are also enriched in PR compared to input (Figure S1D). These observations suggest that isolated PR fractions are not reflective of pure PRs as they are contaminated with other retinal cell types. Examination of steady-state gene expression of PI-kinases and PI phosphatases (primers listed in Tables S2 and S3) indicates that PR has enriched levels of class IA PI3K catalytic subunits (*Pik3ca* and *Pik3cd*), class II PI3K (*Pik3c2b*), class III PI3K (*Pik3c3*), and class IB-PI3Kγ-regulatory subunits (*Pik3r5*) (Figure S2A). No enrichment of PI4Ks but enrichment of PI5Ks (*Pip5k1a, Pip5k2b,* and *Pip5k2c*) in PR (Figures S2B and C). We found enriched levels of PI3-phosphatases (*Mtm1, Mtmr4,* and *Mtmr8*), and PI5-phosphatases (*Synj1, Synj2,* and *Inpp5e*), but not PI4-phosphatases (Figure S2D–F). The mitochondrial phosphatase, *Ptpmt1*, and phosphatidyl inositol-transfer protein-β (*Pitpβ*) are also enriched in PR (Figure S2G).

**Table 1.** Phosphoinositide kinases in mammalian genome.

Class/type	Protein	Gene name	Reaction catalyzed
Class IA PI3K	p110α	*Pik3ca*	**A, D, H**
	p110β	*Pik3cb*	**A, D, H**
	p110δ	*Pik3cd*	**A, D, H**
Regulatory subunits		** **	
	p85α	*Pik3r1*	* *
	p85β	*Pik3r2*	* *
	p85γ	*Pik3r3*	* *
	Vps15	*Pik3r4*	* *
	p101-PI3K	*Pik3r5*	* *
	p87-PI3K-adapter	*Pik3r6*	* *
Class IB PI3K	p110γ	*Pik3cg*	**A, D, H**
Class II PI3K	PI3K-C2α	*Pik3c2a*	**A, D**
	PI3K-C2β	*Pik3c2b*	**A, D**
	PI3K-C2γ	*Pik3c2g*	**A, D**
Class III PI3K	Vps34	*Pik3c3*	**A**
Phosphatidylinositol 4-kinases		** **	* *
Type II PI4Ks	PI4K IIα	*Pi4k2a*	**B**
	PI4K IIβ	*Pi4k2b*	**B**
Type III PI4Ks	PI4K IIIα	*Pi4kca*	**B**
	PI4K IIIβ	*Pi4kcb*	**B**
Phosphatidylinositol phosphate kinases		** **	* *
Type I PIPKs	PIPK 1α	*Pip5k1a*	**F**
	PIPK Iβ	*Pip5k1b*	**F**
	PIPK Iγ	*Pip5k1c*	**F**
Type II PIPKs	PIPK IIα	*Pip4k2a/Pip5k2a*	**G**
	PIPK IIβ	*Pip4k2b/Pip5k2b*	**G**
	PIPK IIγ	*Pip4k2c/Pip5k2c*	**G**
Type III PIPKs	PIPK III	*Pip5k3*	**C, E**
Other		* *	** **
Inositol polyphosphate multikinase	IPMK	*Ipmk*	**H, S, T**

**Table 2.** Phosphoinositide phosphatases in mammalian genome.

Class/type	Protein	Gene name	Reaction catalyzed
Phosphoinositide 3-phosphatases		** **	* *
PTEN	PTEN	*Pten*	**I, R**
Myotubularins	MTM1	*Mtm1*	**I, N**
	MTMR1	*Mtmr1*	**I, N**
	MTMR2	*Mtmr2*	**I, N**
	MTMR3	*Mtmr3*	**I,N**
	MTMR4	*Mtmr4*	**I, N**
	MTMR6	*Mtmr6*	**I, N**
	MTMR7	*Mtmr7*	**I, N**
	MTMR8	*Mtmr8*	**I**
	MTMR14	*Mtmr14*	**I, N**
Phosphoinositide 4-phosphatases		** **	* *
INPP4	INPP4A	*Inpp4a*	**L**
	INPP4B	*Inpp4b*	**L**
TMEM55	TMEM55A	*Tmem55a*	**P**
	TMEM55B	*Tmem55b*	**P**
Phosphoinositide 5-phosphatases		** **	* *
Type II INPP5s	SYNJ1	*Synj1*	**I, J, M, O, P, Q**
	SYNJ2	*Synj2*	**I, J, M, O, P, Q**
	OCRL1	*Ocrl*	**O, Q**
	INPP5B	*Inpp5b*	**O,Q**
	SKIP	*Skip*	**O, Q**
Type IV INPP5	INPP5E	*Inpp5e*	**M, O, Q**
Others		** **	* *
PLIP	PLIP	*Ptpmt1*	**K**
Sac	SAC1	*Sacm1l*	**I, J, M**
	SAC2	*Inpp5f*	**O, Q**
	SAC3	*Fig 4*	**M**

### Characterization and cell-type-specific expression of PI-converting enzymes

To better determine the composition of PI-converting enzymes in the retina, while avoiding the contamination between different retinal cell types, we employed TRAP technology to isolate actively translating mRNAs from a single retinal cell type. Breeding of floxed hemagglutinin-tagged (HA) ribosomal Rpl22 mice with retina cell-specific Cre-drivers resulted in the tagging of the HA epitope to ribosomes in a cell-specific manner. Retinal lysates immunoprecipitated (IP) with HA antibodies facilitate the purification of ribosomes with cell-specific associated mRNAs. qRT-PCR was then performed to provide the accurate quantification of gene expression from various retinal cell types. We recently used the TRAP technique to isolate PI kinases and PI phosphatase signatures from rod photoreceptor cells using rhodopsin-Cre mice mated with HA-tagged Rpl22 mice (26). In this study, we have extended the scope of our study to isolate actively translating mRNAs from cone photoreceptors, Müller, RPE, and ganglion cells, and identified cell-type-specific PIP signatures.

### Interpretation of the data

We described the data in the context of enrichment and depletion, in the manner of previous studies (26, 27), i.e. when a transcript is enriched, there is an increased level of transcript in our HA-TRAP samples when compared to input (total retina), suggesting that a particular cell type contributes a significant source of transcript in the retina. When a transcript is depleted, there is a decreased level of transcript in our HA-TRAP samples when compared to input (total retina), suggesting that a significant source of transcript in the retina does not come from the particular cell type, but other cell types. Depletion of a transcript in a specific cell type does not imply that it is absent but that transcript is predominantly expressed in a different cell type.

### Cone photoreceptor-specific PIP signatures

To isolate cone-specific actively translating mRNAs, we mated HA-tagged Rpl22 mice with mice carrying Cre-recombinase under the control of a human red/green opsin promoter (28). Immunohistochemical analysis with HA antibody shows the activation of HA-tagged ribosomal protein in the cone inner segments (Fig. 2C–E). Polyribosomal IP followed by qRT-PCR analysis with various retina cell-specific primers shows a significant enrichment of cone-specific transcript (cone-opsin, *Opn1sw*), and depletion of all other retinal cell transcripts tested (Fig. 2F). The cone photoreceptors show significant enrichment of catalytic subunits of class IA PI3Ks (*Pik3ca and PIk3cb*), class IB PI3K (*Pik3cg*), class II PI3K (*Pik3c2a* and *PIk3c2b*β), class III PI3K (*Pik3c3*), and regulatory subunits of class IA PI3K (*Pik3r1*) and class II PI3K (*Pik3r*6) (Figure S3A). The class IA PI3K inhibitory protein, *Pik3ip1* was enriched in cone photoreceptor cells (Figure S3A). We found significantly enriched levels of PI4 kinase, *Pi4k2b,* and several PI5 kinases (*Pip5k1a, Pip5k1b, Pip5k2a,* and *Pip5k3*) (Figure S3B, C). The PI3 phosphatases, *Mtmr5, Mtmr6* and *Mtmr8*, PI4 phosphatase, *Tmem55a* and PI5 phosphatases, *Ocrl, Inpp5b, Inpp5e,* and *Skip*, were enriched in cone photoreceptor cells (Figure S3D–F). The mitochondrial PI phosphatase, *Ptpmt1*, the PI phosphatase *Sacm11* that regulates Golgi membrane morphology, *Vac14* (PIKFyve regulatory proteins), and *Pitpβ* are enriched in cones (Figure S3G).

### RPE-specific PIP signatures

**Fig. 3.**
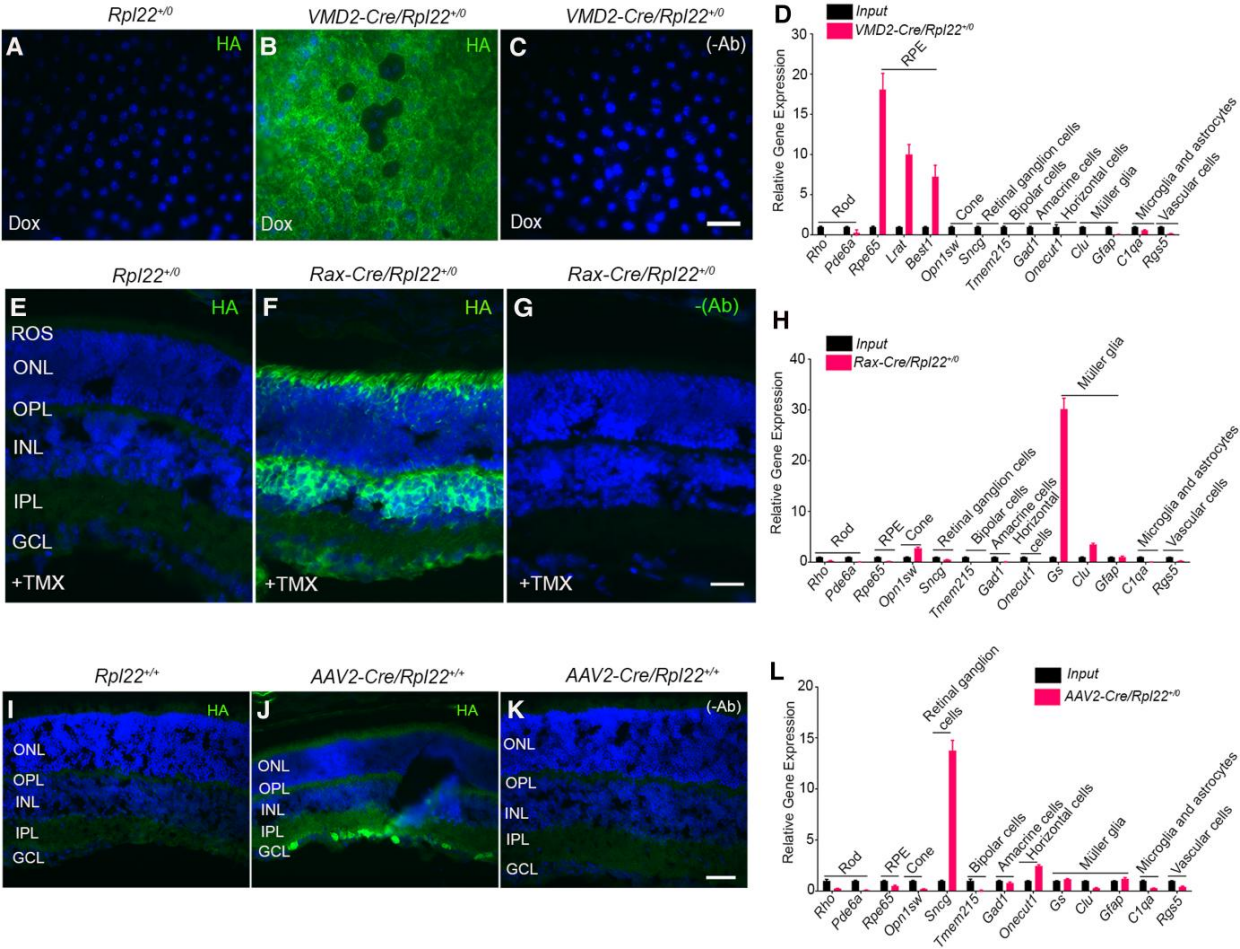
Characterization of VMD2 (RPE)-Cre/Rpl22, Rax-Cre/Rpl22, and AAV2-Cre/Rpl22 mouse lines. Retinal flat mounts from *Rpl22^+/0^* (A), *VMD2-Cre/Rpl22^+/0^* (B) mice were stained with anti-HA antibodies. Omission of HA antibody (C). Equal amounts of mRNA from the RPE + retina (input) and VMD2-Cre IP samples were used to measure retinal cell markers by qPCR and the data normalized to *Rpl37* and *Rpl38* levels (D). Retinal sections from *Rpl22^+/0^* (E), and *Rax-Cre^ER^/Rpl22^+/0^* (F) mice were stained with anti-HA antibodies. Omission of HA antibody (G). Equal amounts of mRNA from the retina (input) and Rax-Cre IP samples were used to measure retinal cell markers by qPCR, and the data normalized to *Rpl37* and *Rpl38* levels (H). Retinal sections from *Rpl22^+/0^* (I), and *AAV2-Cre/Rpl22^+/0^* (J) mice were stained with anti-HA antibodies. Omission of HA antibody (K). Equal amounts of mRNA from the retina (input) and AAV-2-Cre IP samples were used to measure retinal cell markers by qPCR and the data normalized to *Rpl37* and *Rpl38* levels (L). Data mean ± *SEM* (*n = 3*). Note: RPE-Cre has some ectopic expression in the neural retina. Thus, we mechanically separated the RPE from the neural retina and then carried out the polyribosomal IP to isolate actively translating mRNAs from the RPE.

To determine the PIP signatures in RPE cells, we bred doxycycline (Dox)-inducible RPE cell-specific VMD2-Cre mice (29) with HA-tagged-Rpl22 mice. Two doses of doxycycline (Dox, 10 mg) were gavaged every day for 2 days. One week later, RPE flat mounts from VMD2-Cre/Rpl22 mice showed robust expression of HA-tagged Rpl22 (Fig. 3B). RPE flat mounts from Rpl22 mice without Cre did not express HA (Fig. 3A). The omission of HA antibody on the VMD2-Cre/Rpl22 mouse flat mount did not show HA immunoreactivity (Fig. 3C). qRT-PCR analysis of various cell-specific markers shows enrichment of RPE cell markers (*Rpe65, Lrat,* and *Best1*) and depletion of all other retinal cell transcripts (Fig. 3D). The PI enzyme profile indicates enrichment of class IA PI3K regulatory subunit, *Pik3cb* and class II PI3K, *Pik3c2b* and enrichment of regulatory subunit of class I PI3Kγ, *Pik3r6,* and class I PI3K inhibitory protein, *Pik3ip1* (Figure S4A). Concerning PI4K, *Pik4ca*, and PI5K, *Pip5k1b, and Pip5k2c* were enriched in RPE cells (Figure S4B, C). The PI3-phosphatases, *Mtmr1* and *Mtmr3*, PI4-phosphatase, *Tmem55a*, and PI5-phosphatase, *Skip* were enriched in RPE cells (Figure S4D–F). The mitochondrial PI-phosphatase, *Ptpmt1* was also enriched in RPE cells (Figure S4G).

### Müller cell-specific PIP signatures

To evaluate the PIP signatures in Müller cells, we generated Müller cell-specific HA-Rpl22 mice by breeding floxed HA-tagged Rpl22 mice with tamoxifen-inducible Cre mice under the control of Rax promoter (30). The commonly used glial high-affinity glutamate transporter (GLAST)-Cre targets both Müller glia and astrocytes (31). Thus, we used Rax-Cre^ER^, which targets Müller glia but not astrocytes (30). One-month-old mice were orally gavaged with 1 mg tamoxifen (TMX) every alternate day for 3 days. Three weeks later, immunohistochemistry with HA antibody showed robust expression of HA-tagged Rpl22 in TMX-administered mice but not in Rpl22 mice who received peanut oil as a control (Fig. 3E–G). Polyribosomal IP followed by the qRT-PCR analysis of various retinal cell-specific markers shows enrichment of Müller cell markers (glutamine synthetase (*Gs*), and clusterin (*Clu*)) and depletion of all other retinal cell transcripts (Fig. 3H). However, we observed a slight elevation of the cone signal in Rax-Cre. This observation further confirms a previously published study on the expression of Rax in cones (32). Müller cells show enrichment of class III PI3K, *Pik3c3* and class I PI3Kγ regulator, *Pik3r6* (Figure S5A). The PI4-kinase, *Pi4k2b*, and PI5K, *Pip5k1b* are enriched in Müller cells (Figure S5B, C). We found no enrichment of PI3, PI4, and PI5 phosphatases in Müller cells (Figure S5D–G), suggesting that Müller cells might require PI kinase signaling more than PI phosphatase signaling under physiological conditions.

### Ganglion cell-specific PIP signatures

To identify the PIP signatures in RGCs, we have intravitreally injected 1.5 μl of AAV2-Cre virus (33) suspension containing over 5.0 × 10^11^ particles/ml into the eyes of 2-month-old HA-Rpl22 floxed mice. Two weeks after the administration of AAV2-Cre (34), retinal sections were immunoblotted with HA antibody and the results indicate the expression of HA in RGCs in AAV2-Cre-injected mice but not in Rpl22 mice with PBS injection (Fig. 3I–K). Polyribosomal immunoprecipitation followed by the qRT-PCR analysis of various retinal cell-specific markers shows enrichment of retinal ganglion cell marker, gamma synuclein, *Sncg*, and depletion of all other retinal cell transcripts (Fig. 3L). RGCs show enrichment of class IA PI3K catalytic subunits (*Pik3ca, Pik3cb,* and *Pik3cd*), class IB PI3K (*Pik3cg*), class II PI3K (*Pik3c2b* and *Pik3c2g*), the regulatory subunit of class IB PI3K, *Pik3r6* and class I PI3K inhibitory protein, *Pik3ip1* (Fig. S6A). We found no enrichment of PI4Ks, but enrichment of PI5K, *Pip5k2b* in RGCs (Figure S6B, C). In RGCs, the PI3-phosphatase, *Mtmr8* was enriched (Figure S6D). There was no enrichment of PI4-phosphatases, but enrichment of PI5-phosphatase, *Synj2* in RGCs (Figure S6E, F). We also found the enrichment of the PIKFyve regulatory protein, *Vac14* in RGCs (Figure S6G).

### Heatmap analysis of PI-converting enzymes between Opti-prep, rod, and cone cells

The Opti-prep data were analyzed for enrichment and depletion of PIPs (Figure S2). The Volcano plot shows the enriched and depleted genes (Figure S7A), and the principal component analysis (PCA) analysis shows that genes enriched in Opti-prep do not group with any other retinal cell type (Figure S7B); however, they did associate with photoreceptors on the PC1 axis. This suggests the enrichment of distinct genes in the Opti-prep samples not enriched in other retinal cells. Concordantly, the heatmap analysis shows that enriched PI-converting enzyme signatures in Opti-prep are distinct in their expression (Figure S7C), even though, the Opti-prep recovers PRs. This could be due to contamination of other cell types such as amacrine cells, microglia and astrocytes, and vascular cells (Figure S1D). The Venn diagram show enrichment of six genes in Opti-prep, 14 genes in rods, and six genes in cones (Figure S7D, Table S4). Four genes are commonly enriched between Opti-prep and rods (*Pik3c3, Ptpmt1, Mtmr3*, and *Mtmr4*) and 1 gene is commonly enriched between cones and Opti-prep (*Pik3c2b*) (Figure S7D, Table S4). The analysis also revealed six genes are depleted in Opti-prep, six genes in rods, and seven genes in cones (Figure S7E, Table S5). Three of the depleted genes in Opti-prep are common to both rods and Opti-prep (*Pik3r1, Pi4kca,* and *Pik3c2g*) whereas 1 gene in Opti-prep is common to both Opti-prep and cones (*Tmem55b*) (Figure S7E, Table S5). We also found that there is not a common gene that is either enriched or depleted in all three groups. Taken together, this suggests that Opti-prep isolated RNA is not reflective of Rod/Cone gene expression.

### Heatmap analysis of PI-converting enzymes between retinal cells

**Fig. 4.**
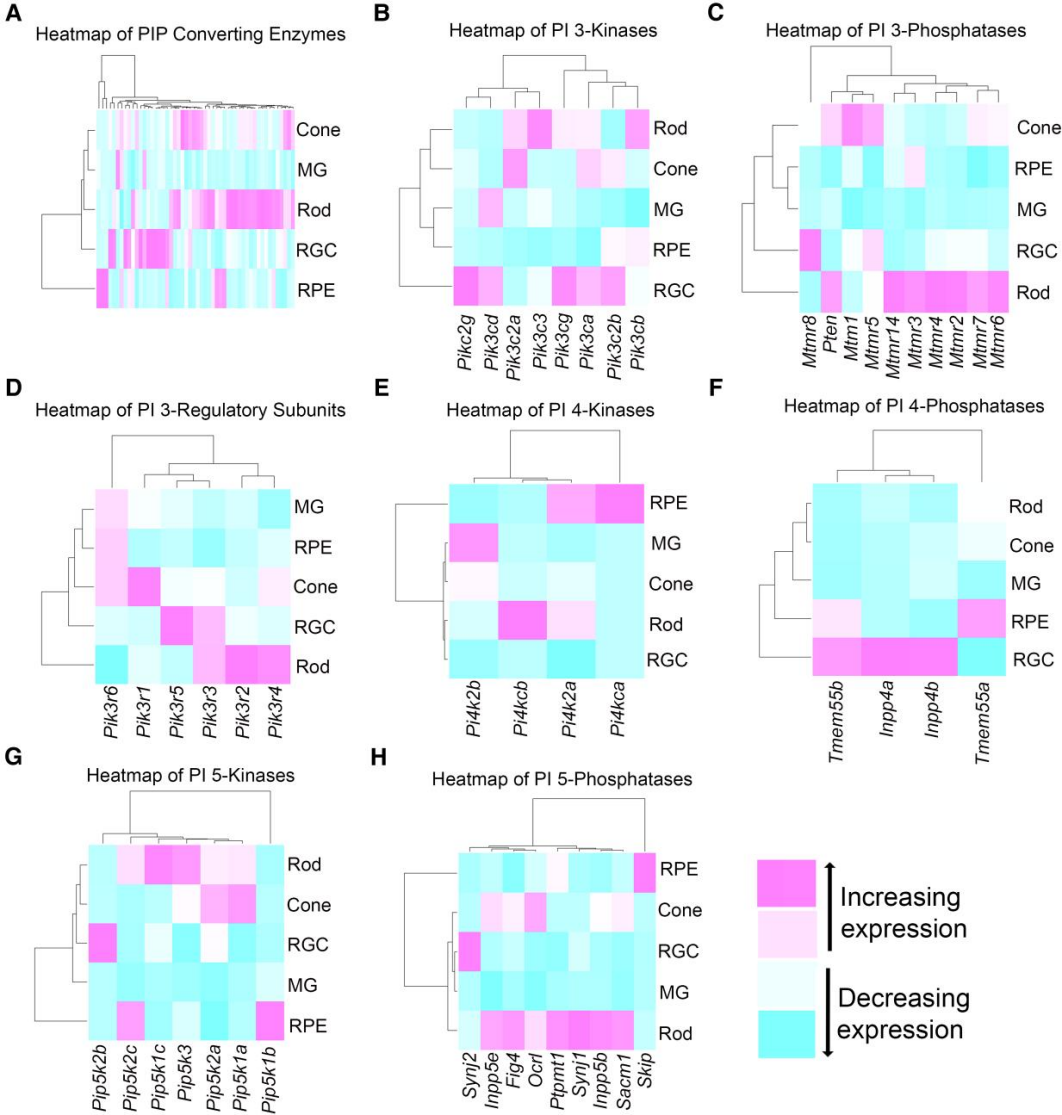
Heatmap showing the color-coded correlation between various retinal cells and PI-converting enzymes. Heatmap of PI-converting enzymes (A), PI 3-kinases (B), PI3-phosphatases (C), PI3-regulatory subunits (D), PI 4-kinases (E), PI 4-phosphatases (F), PI 5-kinases (G), and PI 5-phosphatases (H).

Using results from the qPCR analysis of PI-converting genes between retinal cell types, heat maps were generated to illustrate differential expression between different retinal cell types. Rods, cones, MGs, RGCs, and RPE showed distinct signatures in their expression of PI-converting genes, with rods, RGCs, and cones showing greater enrichment of PI-converting genes, whereas MG and RPE showed less enrichment. This seemingly suggests that rods, cones, and RGCs have dynamic changes in the PIP pools (Fig. 4A). To better identify the composition of these distinct signatures, we subgrouped the heatmaps by PI-converting classes. PI-3 kinases showed greater enrichment in rods and RGCs compared to other retinal cell types (Fig. 4B). Heatmaps of PI-3 phosphatases showed greater enrichment in rods and cones compared to other retinal cell types (Fig. 4C). However, the enriched genes appeared to be mutually exclusive between rods and cones with distinct genes being enriched for rods (*Mtmr13*, *Mtmr3*, *Mtmr4*, *Mtmr2*, and *Mtmr7*) and distinct genes being enriched for cones (*Mtmr1*, *Mtmr5*). PI-3 regulatory subunits showed distinct expression (Fig. 4D), often mutually exclusive expression, of different regulatory subunits between different retinal cell types: Rods (*Pi3kr2, Pi3kr4*), cones (*Pik3r1*), RGCs (*Pik3r5*), MG, and RPE (*Pik3r6*). PI-4 kinases showed increased expression (Fig. 4E) in the RPE (*Pik4ca*, *Pi4k2a*) and Rods (*Pi4k2a*, *Pi4kcb*) followed by a lesser expression in MG and cones (*Pi4k2b*) (Fig. 4E). RGCs did not show enrichment of PI4K genes. Surprisingly, however, most PI-4 phosphatases were enriched in RGCs and not in other retina cell types (Fig. 4F). Both PI-5 kinases and phosphatases, however, showed enrichment in photoreceptors with enrichment in rods dominating followed by enrichment in cones (Fig. 4G and H).

### PCA of PI-converting enzymes between retinal cells

PCA analysis was used to emphasize variation and determine the closeness of PI-converting enzymes in various retinal cell types. PC analysis of the expression of PI-converting enzymes between the retinal cell types showed clustering of MG and RPE. Rods and cones were separated from nonphotoreceptor cells on the PC1 axis (Figure S8A). To better identify the composition of these distinct signatures, we subgrouped the PC plots by PI-converting classes. PC analysis of PI-3 kinases showed rods clustering with cones and separated from MG, and RPE cells, whose clustering was driven by a lack of PI-3 kinase enrichment (Figure S8B). PC analysis of PI-4 kinases showed rod's clustering with cones and driven by their shared expression of *Pi4kcb*, all other retinal cell types were separated suggesting distinct PI-4 kinases expression between different retinal cell types (Figure S8C). PC analysis of PI-5 kinases showed rods clustering with cones and driven by their shared expression of *Pip5k3*, *Pip5k1a*, and *Pip5k1c* all other retinal cell types were separated suggesting distinct PI-5 kinases expression between different retinal cell types (Figure S8D). PC analysis of PI-3 regulatory subunits shows the separation of all retinal cell types suggesting distinct PI-3 regulatory subunits expression between different retinal cell types (Figure S8E) with RPE and MG clustering together, matching heatmap results showing only moderate expression of *Pik3r6* in those retinal cell types (Figure S8E). PC analysis of PI-3 phosphatases shows clustering of RPE and MG, driven by a lack of expression of PI-3 phosphatases, interestingly; however, cones cluster with RGCs and not rods; Rod clustering is driven by the expression of numerous PI-3 phosphatases (Figure S8F). PC analysis of PI-4 phosphatases shows clustering of rods, cones, and MG, driven by a lack of enrichment of PI-4 phosphatases (Figure S8G). RGCs and RPE show separation on the PC1 axis suggesting distinct expression of PI-4 phosphatases between those retinal cell types (Figure S8G). PC analysis of the expression of PI- 5 phosphatases between the retinal cell types showed clustering of MG and RGC, driven by a lack of enrichment of PI- 5 phosphatase genes. Rods and cones were separated from nonphotoreceptor cells on the PC1 axis (Figure S8H) with rods clustering away from cones due to more expression of PI 5 phosphatases in rods.

### Venn diagram analysis of PI-converting enzymes between retinal cells

**Fig. 5.**
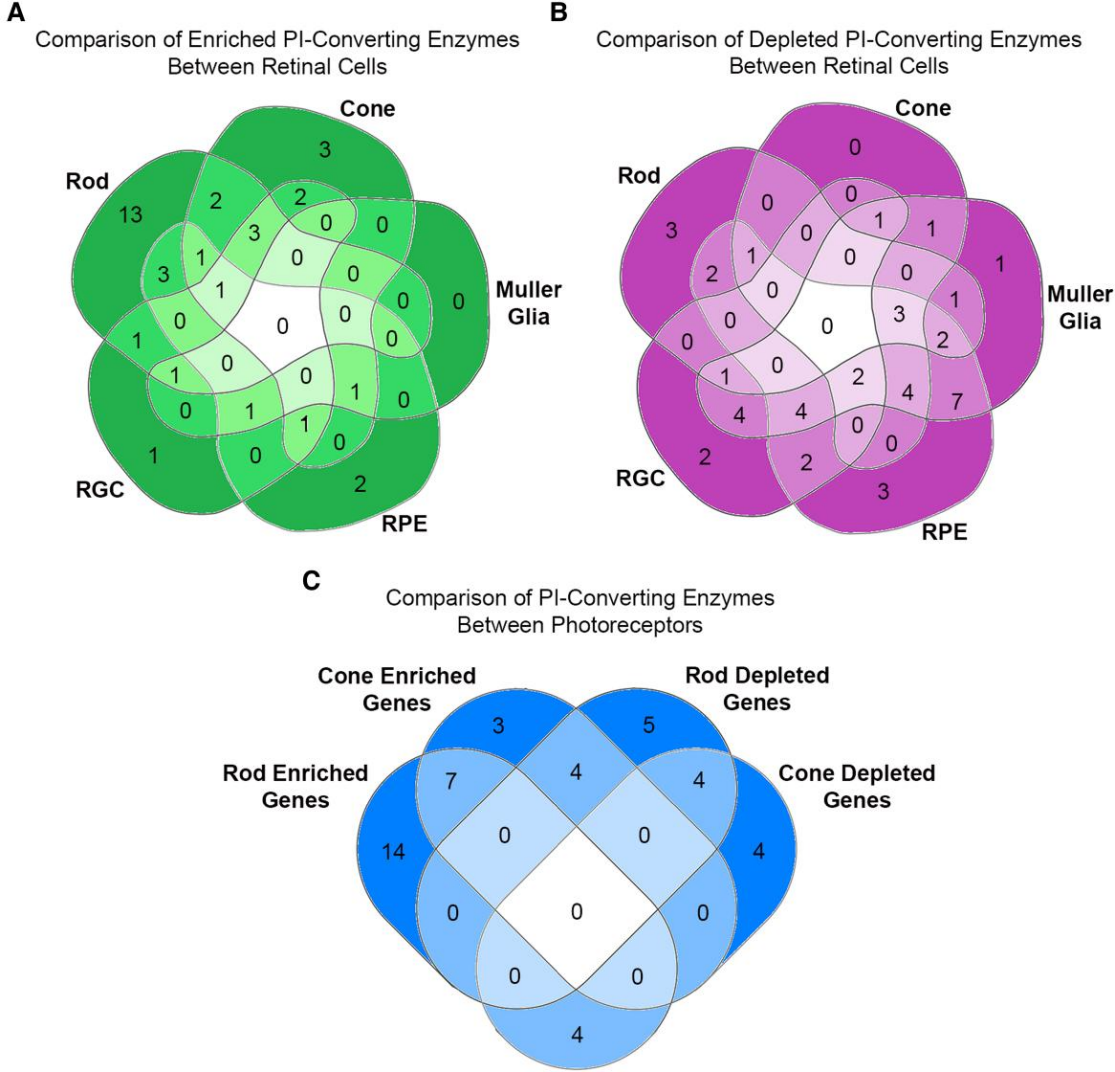
Venn diagram analysis of PI-converting enzymes between retinal cells. Using results from the qPCR analysis, PI-converting genes with significant changes (*P* < 0.05 and log_2_FC > 0.6) were tabulated by whether they were enriched or depleted. Venn diagrams were then created using an online plugin (https://bioinformatics.psb.ugent.be/webtools/Venn/). Comparisons were made between enrichment (A) and depletion (B) of PI-converting enzymes between all retinal cell types. We generated a Venn diagram to identify (1) cone-rich transcripts, which were significantly enriched in the cones; (2) cone-poor transcripts, which were significantly decreased in the cones; (3) rod-rich transcripts, which were significantly enriched in *Rpl22* isolates; and (4) rod-poor transcripts, which were significantly decreased in *Rpl22* isolates (C).

Using results from volcano plots (Figure S9), PI-converting genes with significant changes (*P* < 0.05 and Log_2_FC > 0.6) were tabulated by whether they were enriched or depleted. Venn diagrams were then created using an online plugin (https://bioinformatics.psb.ugent.be/webtools/Venn/). Comparisons were made between the enrichment (Fig. 5A, Table S6) and depletion (Fig. 5B, Table S7) of PI-converting enzymes between all retinal cell types. No single PI-converting enzyme was enriched in all retinal cell types further supporting the distinct expression of PI-converting enzymes between retinal cell types. Additionally, 13 specific genes were enriched in just rods compared to the other retinal cell types. A Venn diagram was generated (Fig. 5C) comparing the rod-rich, cone-rich, rod-poor, and cone-poor transcripts according to the methodology we published previously (26). We also identified differentially expressed PI-converting enzymes between cones and rods and the total retina (Table S8). Using this analysis, we were able to determine that the PI-converting enzymes *Synj1, Pik3r2, Mtmr4,* and *Pi4kcb* are specific for rods, whereas the enzymes *Pik3ip1, Pik3r1, Mtmr8,* and *Pip5k1b* are specific for cones. Additionally, the PI-converting enzymes (*Sacm1, Skip, Pik3cb, Pik3ca, Ipmk, Pip5k2a,* and *Vac14)* were found to be enriched in both rods and cones suggesting that these are PR-specific PI-converting enzymes.

## Discussion

The technique scRNAseq is a well-accepted methodology to identify gene regulatory networks; however, the technique induces variations in gene expression as a result of tissue disruption and delays through cell sorting. In this study, we employed an innovative and novel in vivo TRAP technology (35) to overcome the limitations of scRNAseq and identified retinal cell-type-specific PI-converting enzymes that regulate PI metabolism. Thus far, there is not a single study available on single-cell proteomics that has the depth to identify every protein in a given cell. The TRAP technique we used provides the sensitivity of a bulk RNAseq (high resolution) and the specificity of a scRNAseq. Moreover, the TRAP isolates actively translating mRNAs that are primed to translate to a functional protein. We believe that the TRAP technique is closest to single-cell proteomics, which connects the breach between RNA and protein. The other major advantage is that TRAP assays transcripts from the total population of Cre-positive cells. This will allow us to detect transcript changes in a single-cell type, which may be undetectable in scRNAseq.

Other limitations of scRNAseq include its inefficiency in identifying the total transcriptomes of minor cell populations, e.g. we provide an example of this with RGCs. Studies have shown that when using scRNAseq, each cell provides reads on 10–20% of the total transcriptome. Assuming these reads are random, we can generate a recursive algorithm to model the number of cells required to identify the complete transcriptomic landscape of a cell population. We have modeled cases for 5–20% sampling rates (Figure S10). Assuming a sampling rate of 10% (36), approximately fifty cells need to be read to cover 99.5% of the transcriptional profile for any given cell type. However, this would require at least 25,000 retinal cells to be read to generate a complete transcriptomic profile on RGCs which constitute 0.19% of retinal cells. The scRNAseq approach is much more inefficient compared to TRAP which can isolate the complete transcriptional landscape of RGCs, by isolating all RGC mRNA in a Cre-dependent manner.

In our previous study, we identified the PIP signatures in rods with TRAP technology taking the advantage of rhodopsin-Cre (26). In this study, we have identified PIP signatures using retina cell-specific Cre lines targeted to the cone, RPE, Müller glia, and ganglion cells and compared their expression profiles with each other. This is the first study that examined the expression of PIP signatures in vivo in various retinal cell types. Heatmap analysis revealed that PI-converting enzymes are differentially expressed between different retinal cell types. Interestingly, rods, cones, and RGCs show greater enrichment of PI-converting enzymes whereas less enrichment of these enzymes in Müller glia and RPE. These observations suggest dynamic changes in the PI pools in rods, cones, and RGCs. This higher enrichment of PI-converting enzymes could also be due to the light-sensing activity of rod cones and ganglion cells, especially, intrinsically photosensitive retinal ganglion cells (ipRGCs).

The PI3 kinases are greatly enriched in rods and RGCs compared to other retinal cell types. Interestingly, PI-3 phosphatases also showed greater enrichment in rods and cones compared to other cell types. However, distinct PI-3 phosphatase signatures are observed in rods versus cones. The presence of both PI3 kinases and phosphatases in rods suggests a dynamic active interconversion of one phosphoinositide to another form. These observations also raise an important question why do rods need more PI 3-phosphatases? Do these phosphatases have redundant or nonredundant functions in health and disease? Interestingly, the enriched PI3-phosphatases, especially myotubularin-related phosphatases (*MTMRs*) in rods, are distinct from the cones, suggesting a nonredundant nature of these phosphatases in these cell types. The *Mtmr*-phosphatases are known to regulate autophagy and programmed cell death and they dephosphorylate P(3)P to PI and PI (3,5)P_2_ to PI(5)P (37). The lipid PI(3)P is involved in early endosome biogenesis, whereas PI(3,5)P_2_ is involved in the late endosome and lysosome biogenesis (7). We and others have previously reported that deletion of class III PI3K, Vps34 which generates PI to PI(3)P results in both rod and cone degeneration (38, 39). These observations suggest that PI3-phosphatase regulation is critical for photoreceptor cell survival. The increased enrichment of PI3 kinases and absence of PI3 phosphatases in RGCs suggest that these cells might require sustained PI3K signaling. Further studies are needed to establish these observations.

The PI4-kinases are enriched in RPE and rods and have a lesser expression in Müller glia. The PI4-kinase phosphorylates PI to PI(4)P and it is a substrate for the generation of PI(4,5)P_2_ (40). We recently reported that PI(4,5)P_2_ plays an important role in regulating apical polarity and EGFR endocytosis in RPE (41). In bovine RPE cells, PI(4,5)P_2_ has been shown to regulate the inwardly rectifying potassium channels (42). In the RPE, we observed the enrichment of *Pip5k1b* and *Pip5k2b,* and both are involved in the generation of PI(4,5)P_2_. Interestingly, PI5P4Kα and β express abundant amounts in epiretinal membranes from proliferative vitreoretinopathy (PVR) patients (43). PVR is prevented by the suppression of PI5P4Kα and β enzymes (43). Enhanced expression of these kinases was also observed by the addition of vitreous fluid to RPE cells (43). Even though these kinases are not enriched in our study in the RPE, PVR enhanced these kinases suggesting that PI metabolism is altered in retinal diseases. In RGCs, there was no enrichment of PI4-kinases, but PI4-phosphatases were enriched, suggesting that the dephosphorylation state of PI4-phosphoinositides may promote RGC survival. On the other hand, both PI5-kinases and PI5-phosphatases were not enriched in RGCs, but the enrichment was dominating in rods followed by cones. In rods, PI5-kinase, PIKFyve is enriched which phosphorylates PI and PI(3)P to generate PI(5)P and PI(3,5)P_2,_ respectively (44). Mutations that cause defects in the biosynthesis of PI(5)P and PI(3,5)P_2_ are linked to human diseases including neurodegenerative disorders (44). In the eye, disruption of PIKFyve causes congenital cataracts in humans and zebrafish (45). The Pip5k1c (PIPK Iα) is enriched in rods, and it phosphorylates PI(4)P to PI(4,5)P_2_ (3). Earlier studies show that PI(4,5)P_2_ reporter is localized to rod inner segments and synapses, whereas PI(4)P reporter is localized to outer segments and inner segments (46). Cone photoreceptor enriched with both type I (*Pip5k1a*) and type II (*Pip5k3a*) phosphoinositol phosphate kinases. Type I phosphorylates PI(4)P to generate PI(4,5)P_2_ whereas type II phosphorylates PI(5)P to generate PI(4,5)P_2_ (3).

Mutations in PI 5-phosphatase, OCRL are associated with oculocerebrorenal syndrome of Lowe, a ciliopathy (14, 15). OCRL patients also develop both congenital cataracts and congenital glaucoma (16). Glaucoma affects the optic nerve and results in vision loss due to increased ocular pressure in the eye (47). It has been shown that optogenetic stimulation of OCRL increases aqueous humor and lower IOP in vivo (17, 18). In our study, we did not observe any enrichment of OCRL in RGCs, suggesting that mutations in this gene could be affecting the PI metabolism in these cells. It has also been shown that feeder vessels control IOP (48), and possibly that somatic mutation may not affect the RGCs directly, and changes in PI metabolism may have secondary effects. Further studies are needed to examine the expression of this phosphatase in lymphatic or endothelial cells around the Schlemm's canal. Inositol polyphosphate multikinase (IPMK) belongs to the inositol hexakisphosphate (IP_6_) family of kinases which converts IP_3_ to IP_4_ and IP_4_ to IP_5_ besides containing PI3-kinase activity, phosphorylates PI(4,5)P_2_ to PI(3,4,5) P_3_ (49). However, there are no studies to data on this kinase in the retina. We found the enrichment of this kinase in both rods and cones. Further studies are needed to identify the functional role of this kinase in photoreceptor cells.

**Fig. 6.**
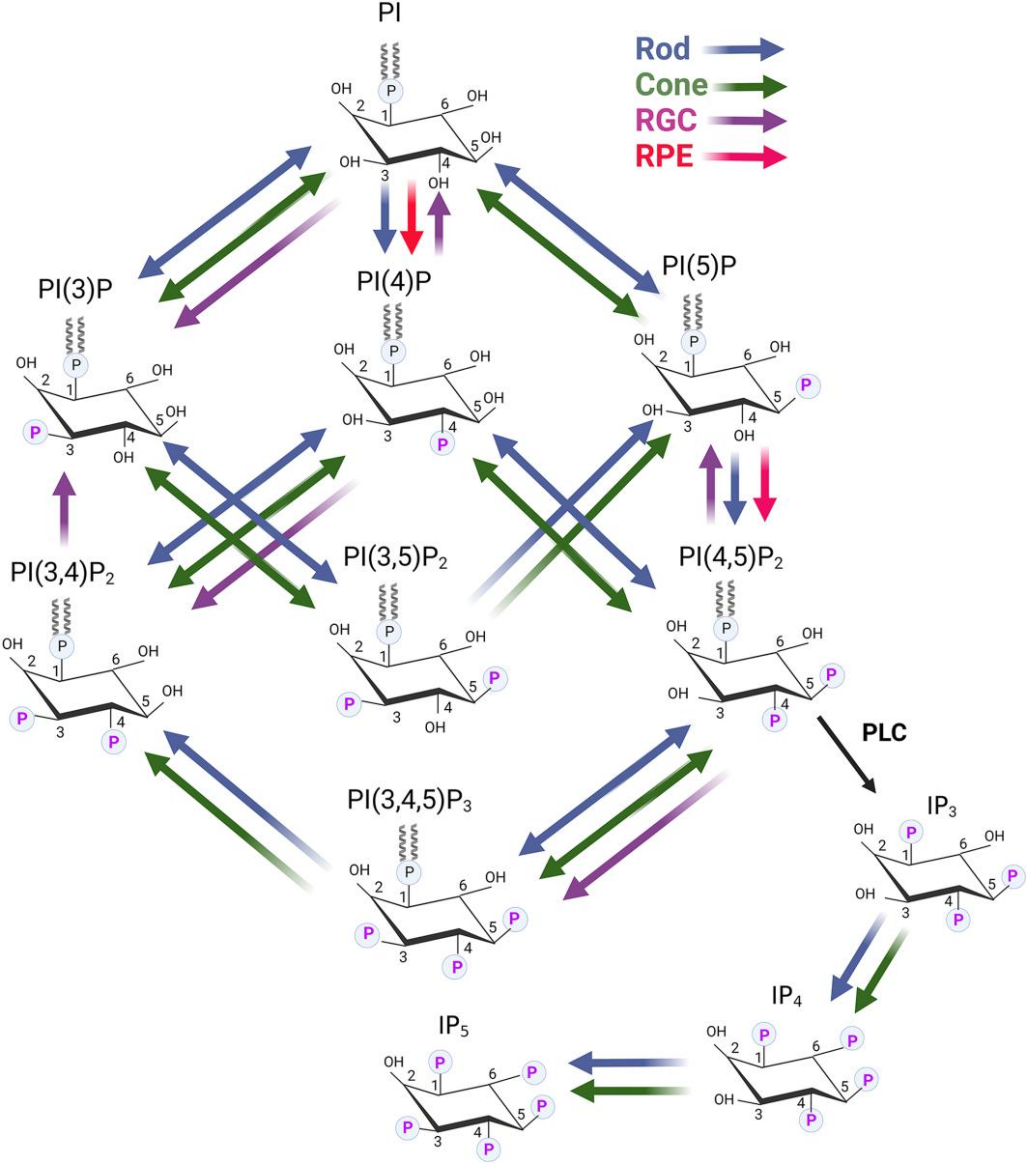
Summary of the current study. In rods and cones, there is the enrichment of PI3-kinases and phosphatases (double-headed arrow) as well as PI5-kinases and phosphatases. Rods also show the enrichment of PI4-Kinases. In RGCs, there is the enrichment of PI3-kinases but not PI3-phosphatases. RGCs also show enrichment of PI4-phosphatases. The RPE shows the enrichment of PI4-kinases. This figure was created with BioRender.com.

In summary, our studies show an active PI metabolism in rod, cone, RGC, and RPE. In rods and cones, PI3-kinase and phosphatase signaling is predominant (Fig. 6). In RGCs, PI3-kinase signaling is predominant but not PI3-phosphatase signaling (Fig. 6). However, PI4-phosphatase signaling is predominant in RGCs (Fig. 6). In RPE, PI4-and PI5-kinase signaling is predominant (Fig. 6). Future studies will include the use of TRAP in disease models to identify how PI metabolism is altered or dysfunctional in pathology.

### Limitations of this study

Although the TRAP technique is suited to isolate actively translating mRNA from single-cell types, it is only as good as the Cre, which is used. Other members in the field have extensively characterized the Cre-drivers we used in our study. However, this technique is extremely sensitive and we were able to detect a minute level of expression of nontarget cell markers with some of these Cre-lines. However, all PIP signatures were distinct from each other suggesting that the majority population, which contributed to the formation of these signatures, belong to the intended cell type. Additionally, this technique allows for rapid and rigorous validation of Cre lines, which we hope other investigators in the field will find useful moving forward. Given the ubiquitous use of Cre-lox to generate retinal KO mice, this information would be of high value to mammalian retinal researchers. Additionally, this study focused on the enrichment and depletion of PI-converting enzymes in the retina; however, lack of expression may not always correlate with the importance of expression. A low-expression protein when absent may be deleterious for the function of a cell. However, using this approach, we were able to generate an atlas of PI-converting enzyme expression between retinal cell types, which we hope will act as a reference and guide future studies in the retina concerning PI-converting enzymes.

## Materials and methods

### Animals

All animal work was conducted according to the *ARVO Statement for the Use of Animals in Ophthalmic and Vision Research* and the *NIH Guide for the Care and Use of Laboratory Animals.* The IACUC at the University of Oklahoma Health Sciences Center has approved all the protocols. Rpl22-RiboTag mice and tamoxifen-inducible RaxCre^ER^ mice were purchased from The Jackson Laboratory (Bar Harbor, Maine). We have described rhodopsin-Cre (26), and human red/green cone-opsin-Cre (28) mice earlier. Tetracycline-inducible VMD2-Cre mice and human red/green cone-opsin promoter-Cre mice were kindly provided by Dr. Yun Le (OUHSC). AAV2-Cre was used to target ganglion cells (CMV packaged into an AAV vector, Vector Biolabs). The mice used in this study were negative for *rd1* and *rd8* mutations. The isolation of retinas for RNA and eyes for immunohistochemistry was carried out as described earlier (26).

### Isolation of polyribosomes containing actively translating mRNAs and quantitative real-time reverse transcription polymerase chain reaction

We isolated polyribosomes containing actively translating mRNAs according to the methodology described earlier (26, 27). The quantitative real-time PCR (qPCR) was carried out as described earlier (26) with the primers (retina cell-specific markers and PI-converting enzymes) listed in Tables S1, S2, and S3. The mRNA levels were normalized by the expression of ribosomal genes *Rpl37* and *Rpl38*. These genes were chosen because the TRAP technique is based on the pull-down of polyribosomes from single cells in a Cre-dependent manner. Therefore, the composition of ribosomal proteins is less likely to be variable between cell types and the total tissues as has been shown in Kouadjo et al. (50). Furthermore, we examined four housekeeping genes actin, *Rpl37*, *Rpl38*, and eukaryotic initiation factor 3 subunit E (*Eif3e*) expression between various retinal cell types. Our results indicate that *Rpl37* and *Rpl38* exhibited less variability between retinal cell types compared to actin and *Eif3e* (Figure S11).

### Statistical significance

Using results from the qPCR analysis, PI-converting genes with significant changes (*P* < 0.05 & log_2_FC > 0.6) were calculated by their enrichment or depletion. All TRAP sample results were normalized to the retina. RPE samples were normalized to (RPE + Retina), and for comparisons against retinal cells, the values were rescaled to be comparable to the retinal samples. All results were tabulated and saved as a CSV file (Supplemental File 1).

## Supplementary Material

pgad063_Supplementary_Data

## Data Availability

All data are included in this article and/or Supplementary material.
