# Supplementary material for: Atlas of phosphoinositide signatures in the retina identifies heterogeneity between cell types
Source: PNAS Nexus. 2023 Mar 3;2(3):pgad063. doi: 10.1093/pnasnexus/pgad063 (PMC10062291; doi:10.1093/pnasnexus/pgad063)
Supplement: pgad063_Supplementary_Data [file pgad063_supplementary_data.zip › PNASNEXUS-PNASNEXUS-2023-00064-T-s01.pdf]

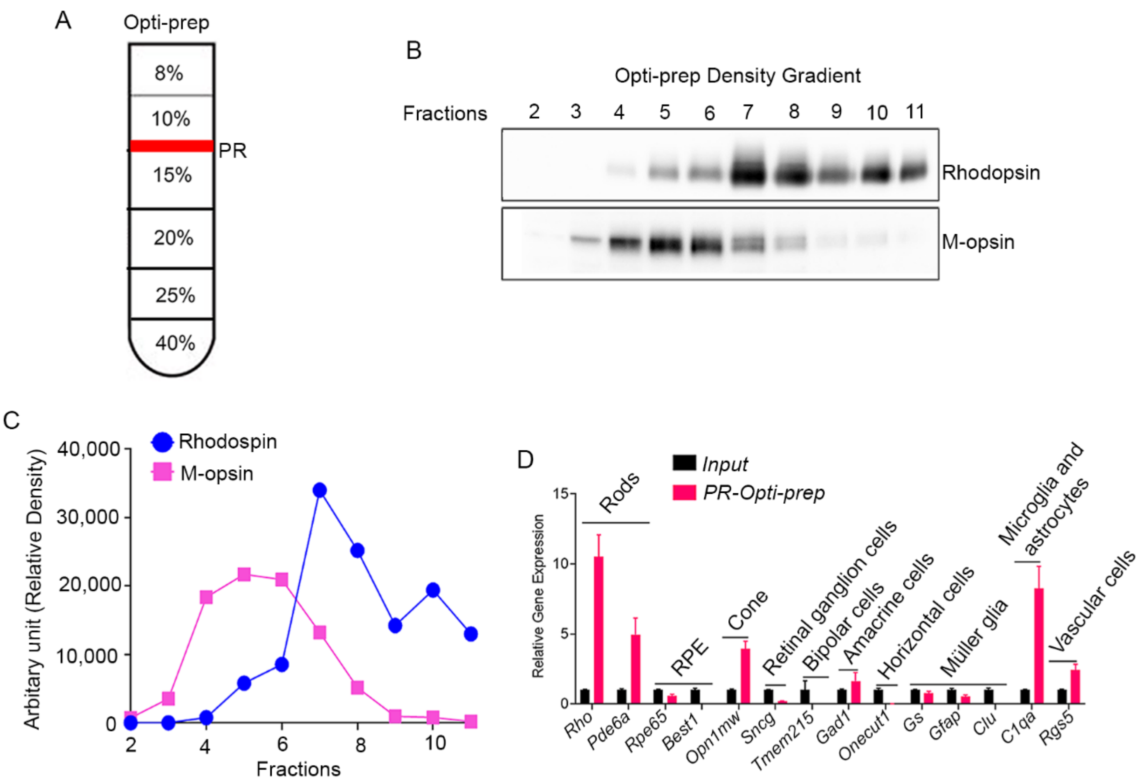


**Figure S1. Isolation and characterization of mouse photoreceptors.** Crude retina lysate was subjected to 8-40% Opti-prep density gradient centrifugation (**A**). Fractions from top to bottom were immunoblotted with rhodopsin and M-opsin antibodies (**B**). Co-elution of rods and cones on Opti-prep gradient centrifugation (**C**). Peak rhodopsin fraction (#7, interface between 10 and 15%) was used to prepare RNA and qPCR analysis was carried out with various retinal cell-specific markers (**D**): Rod (*Rho*, *Pde6a*), retinal pigment epithelium (*Rpe65, Best1*), cone (*Opn1mw*), retinal ganglion cells (*Sncg*), bipolar cells (*Tmem215*), amacrine cells (*Gad1*), horizontal cells (*Onecut1*), Müller glia (*Gs, Gfap, Clu*), microglia and astrocytes (*C1qa*), and vascular cells (*Rgs5*). Data mean ± *SEM* (*n=3*). PR, photoreceptor.

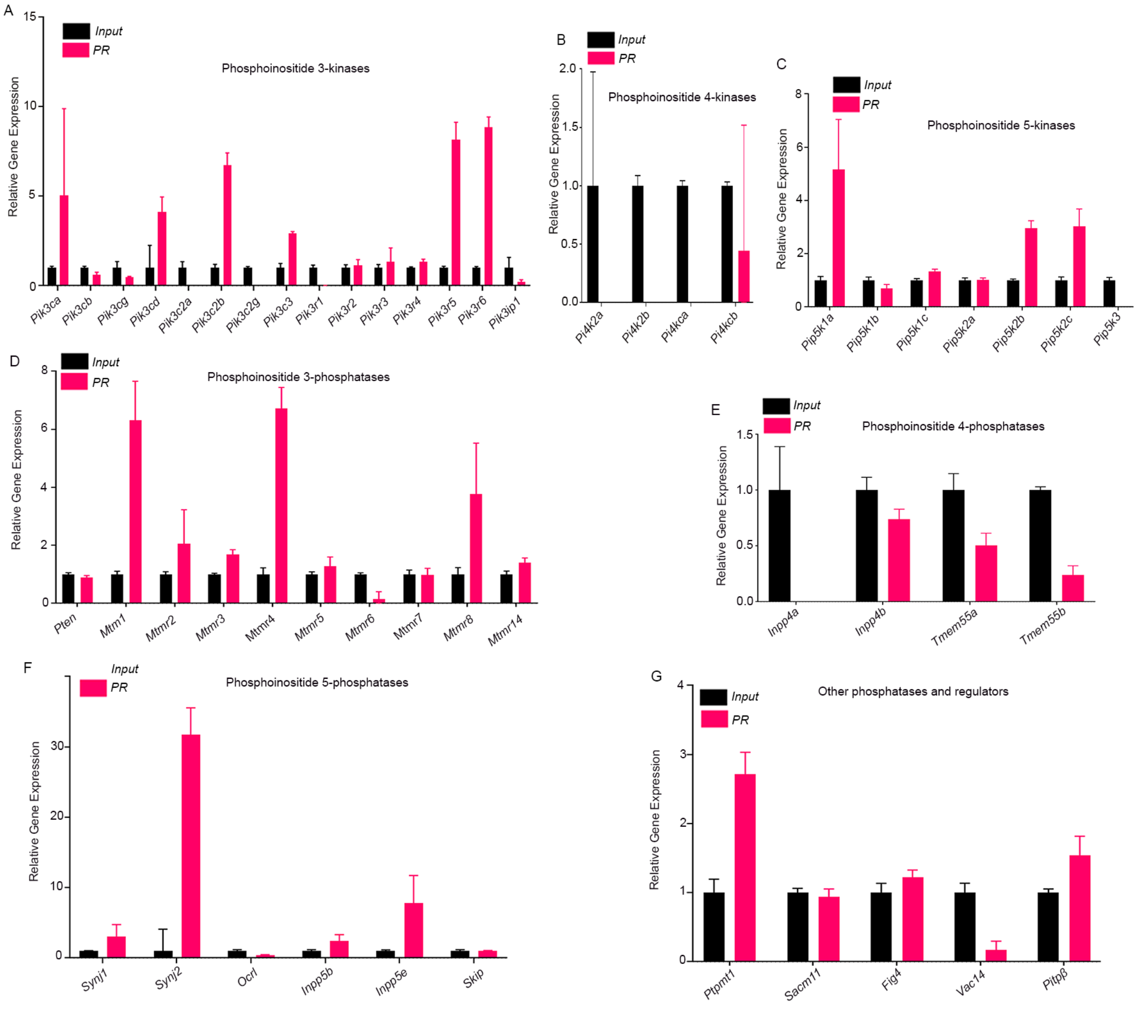


**Figure S2. Gene expression of phosphoinositide kinases and phosphoinositide phosphatases in the Opti-prep samples.** Equal amounts of mRNA from the retina (input), and photoreceptors (PR) isolated by Opti-prep were used for qPCR, and the data normalized to *Rpl37* and *Rpl38* levels. PI-converting enzyme data are presented as phosphoinositide 3-kinases (**A**), phosphoinositide 4-kinases (**B**), phosphoinositide 5-kinases (**C**), phosphoinositide 3-phosphatases (**D**), phosphoinositide 4-phosphatases (**E**), phosphoinositide 5-phosphatases (**F**), and other phosphatases and regulators (**G**). Data are mean ± *SEM* (*n* = 3).

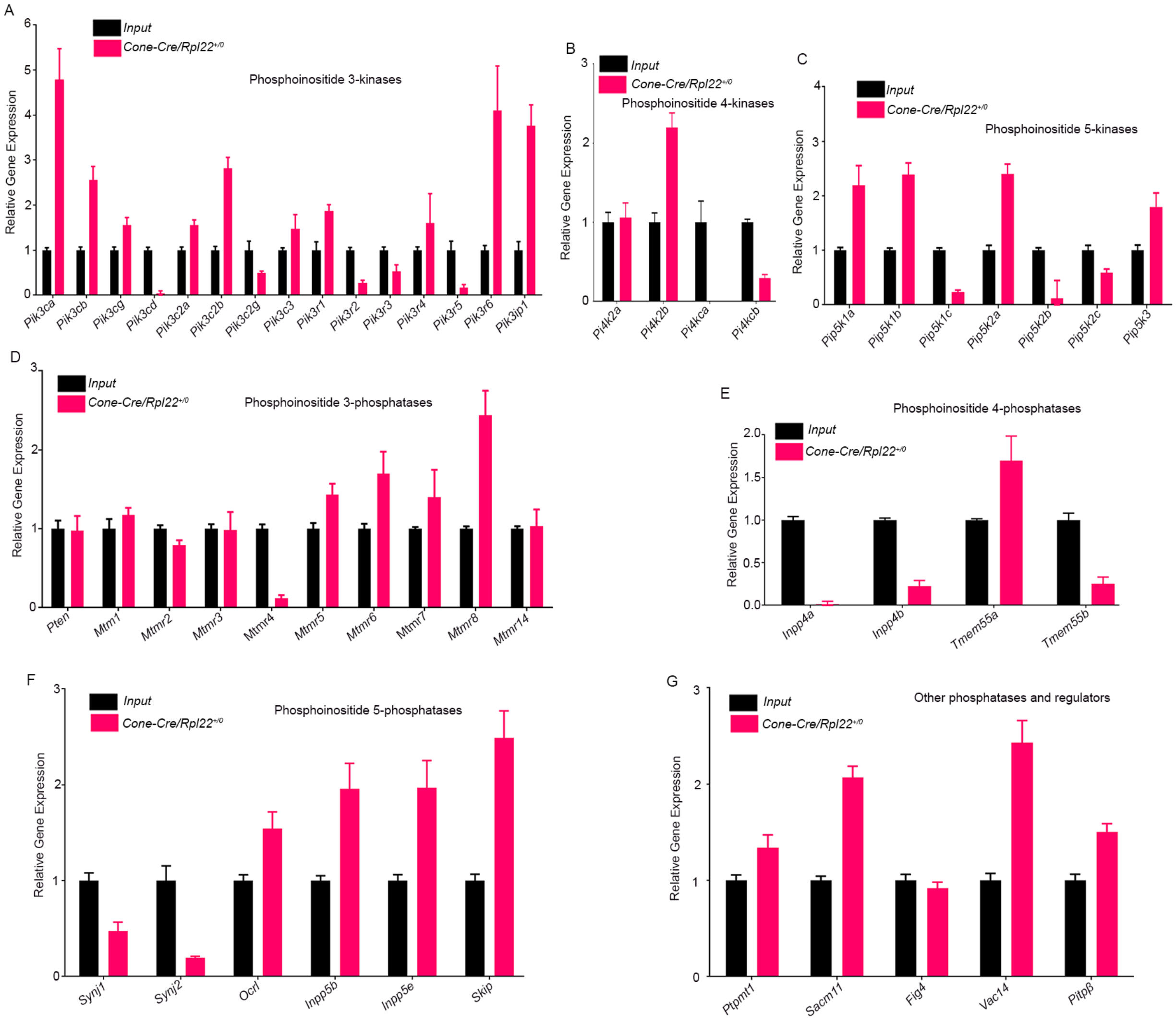


**Figure S3. Gene expression of phosphoinositide kinases and phosphoinositide phosphatases in the cones.** Equal amounts of mRNA from the retina (input), and HA-cone-IP were used for qPCR, and the data normalized to *Rpl37* and *Rpl38* levels. PI-converting enzyme data are presented as phosphoinositide 3-kinases (**A**), phosphoinositide 4-kinases (**B**), phosphoinositide 5-kinases (**C**), phosphoinositide 3-phosphatases (**D**), phosphoinositide 4-phosphatases (**E**), phosphoinositide 5-phosphatases (**F**), and other phosphatases and regulators (**G**). Data are mean ± *SEM (n* = 3).

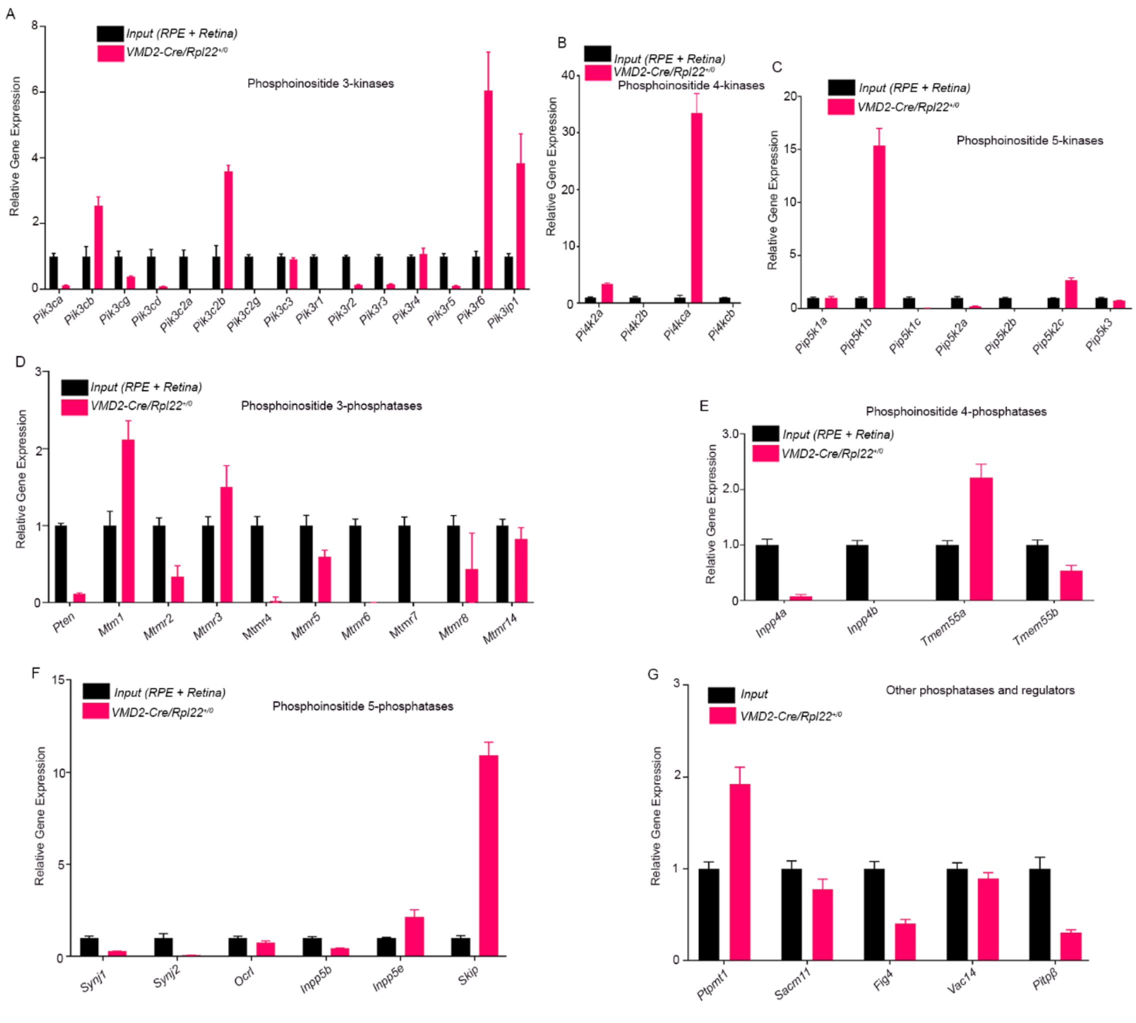


**Figure S4. Gene expression of phosphoinositide kinases and phosphoinositide phosphatases in the RPE.** Equal amounts of mRNA from the retina + RPE (input), and HA-RPE-IP were used for qPCR, and the data normalized to *Rpl37* and *Rpl38* levels. PI-converting enzyme data are presented as phosphoinositide 3-kinases (**A**), phosphoinositide 4-kinases (**B**), phosphoinositide 5-kinases (**C**), phosphoinositide 3-phosphatases (**D**), phosphoinositide 4-phosphatases (**E**), phosphoinositide 5-phosphatases (**F**), and other phosphatases and regulators (**G**). Data are mean ± *SEM* (*n* = 3).

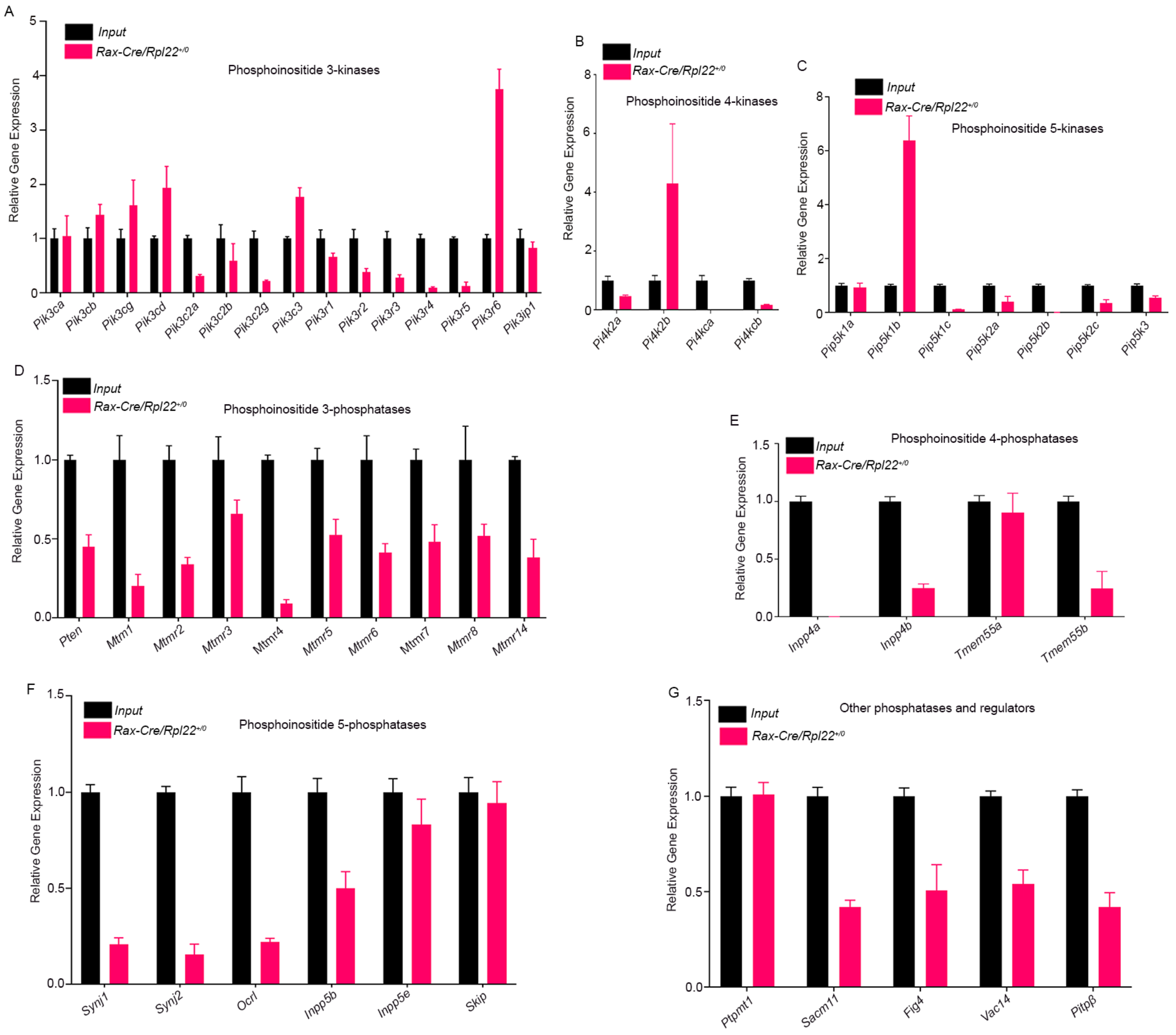


**Figure S5. Gene expression of phosphoinositide kinases and phosphoinositide phosphatases in the Müller cells.** Equal amounts of mRNA from the retina and HA-Müller cell-IP were used for qPCR and the data normalized to *Rpl37* and *Rpl38* levels. PI-converting enzyme data are presented as phosphoinositide 3-kinases (**A**), phosphoinositide 4-kinases (**B**), phosphoinositide 5-kinases (**C**), phosphoinositide 3-phosphatases (**D**), phosphoinositide 4-phosphatases (**E**), phosphoinositide 5-phosphatases (**F**), and other phosphatases and regulators (**G**). Data are mean ± *SEM* ($n$ = 3).

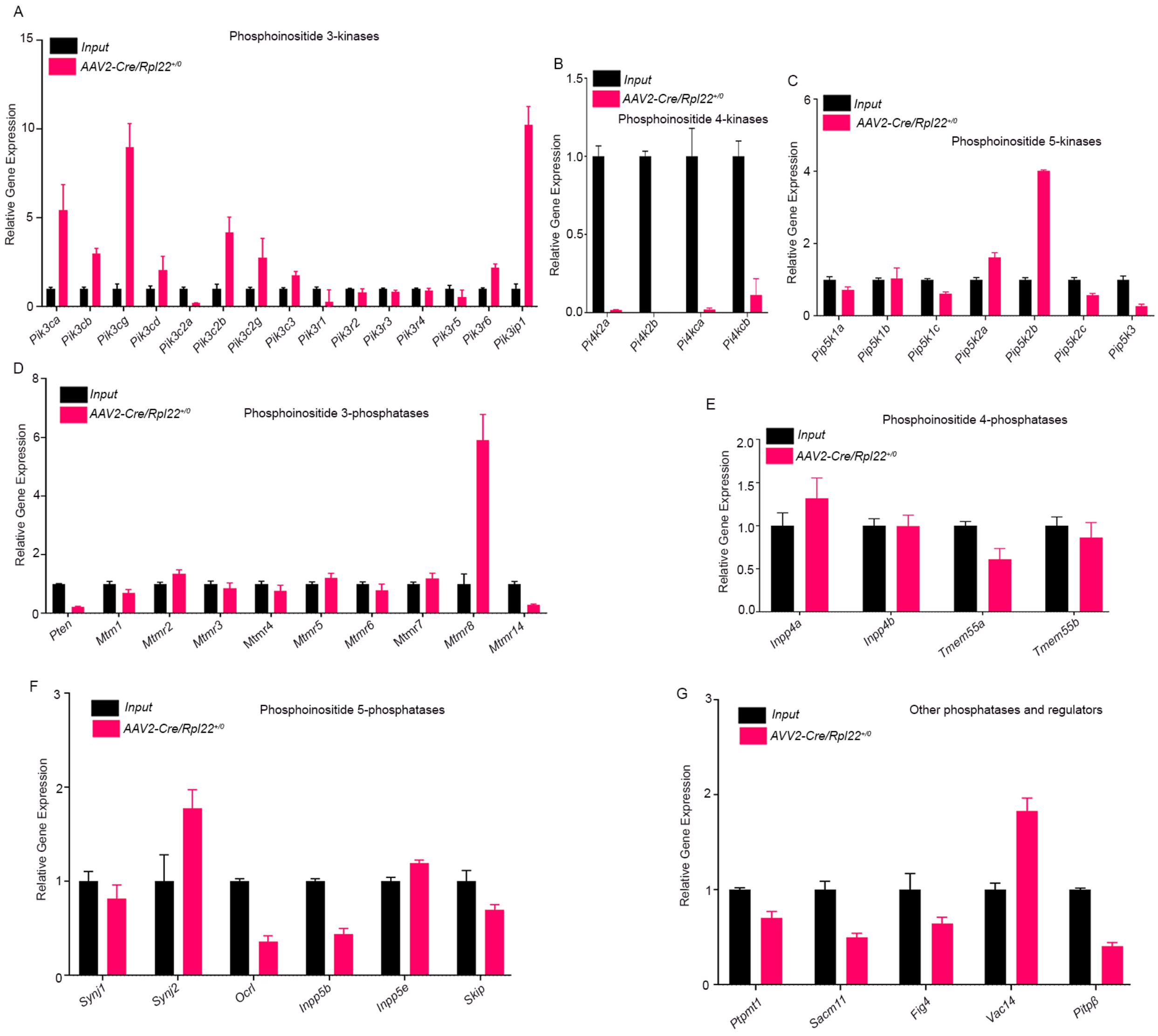


**Figure S6. Gene expression of phosphoinositide kinases and phosphoinositide phosphatases in the retinal ganglion cells.** Equal amounts of mRNA from the retina (input), and HA-ganglion cells-IP were used for qPCR, and the data normalized to *Rpl37* and *Rpl38* levels. PI-converting enzyme data are presented as phosphoinositide 3-kinases (**A**), phosphoinositide 4-kinases (**B**), phosphoinositide 5-kinases (**C**), phosphoinositide 3-phosphatases (**D**), phosphoinositide 4-phosphatases (**E**), phosphoinositide 5-phosphatases (**F**), and other phosphatase and regulators (**G**). Data are mean ± *SEM* (*n* = 3).

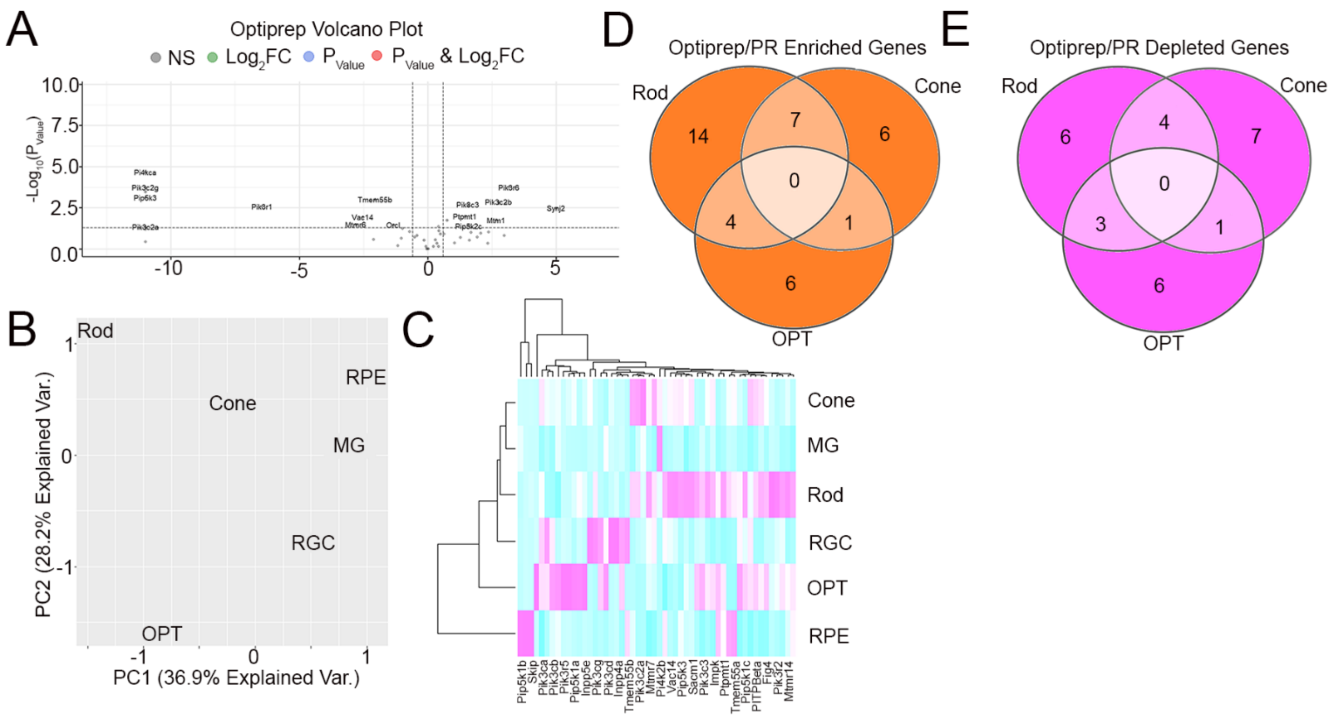


**Figure S7. Comparison of PI-converting enzymes from Opti-prep with rods, cones, and other retinal cell types.** From the data presented in **Fig. S2,** we generated a volcano plot (**A**). The plot is divided into three main categories: bottom, no significance; left upper, significantly increased compared to input; the right upper significantly decreased compared to input. PCA analysis between Opti-prep and various retinal cell types (**B**). Heatmap of PI-converting enzymes between Opti-prep and various retinal cell types (**C**). Venn diagram shows the Opti-prep and photoreceptor (PR) enriched (**D**) and depleted genes (**E**).

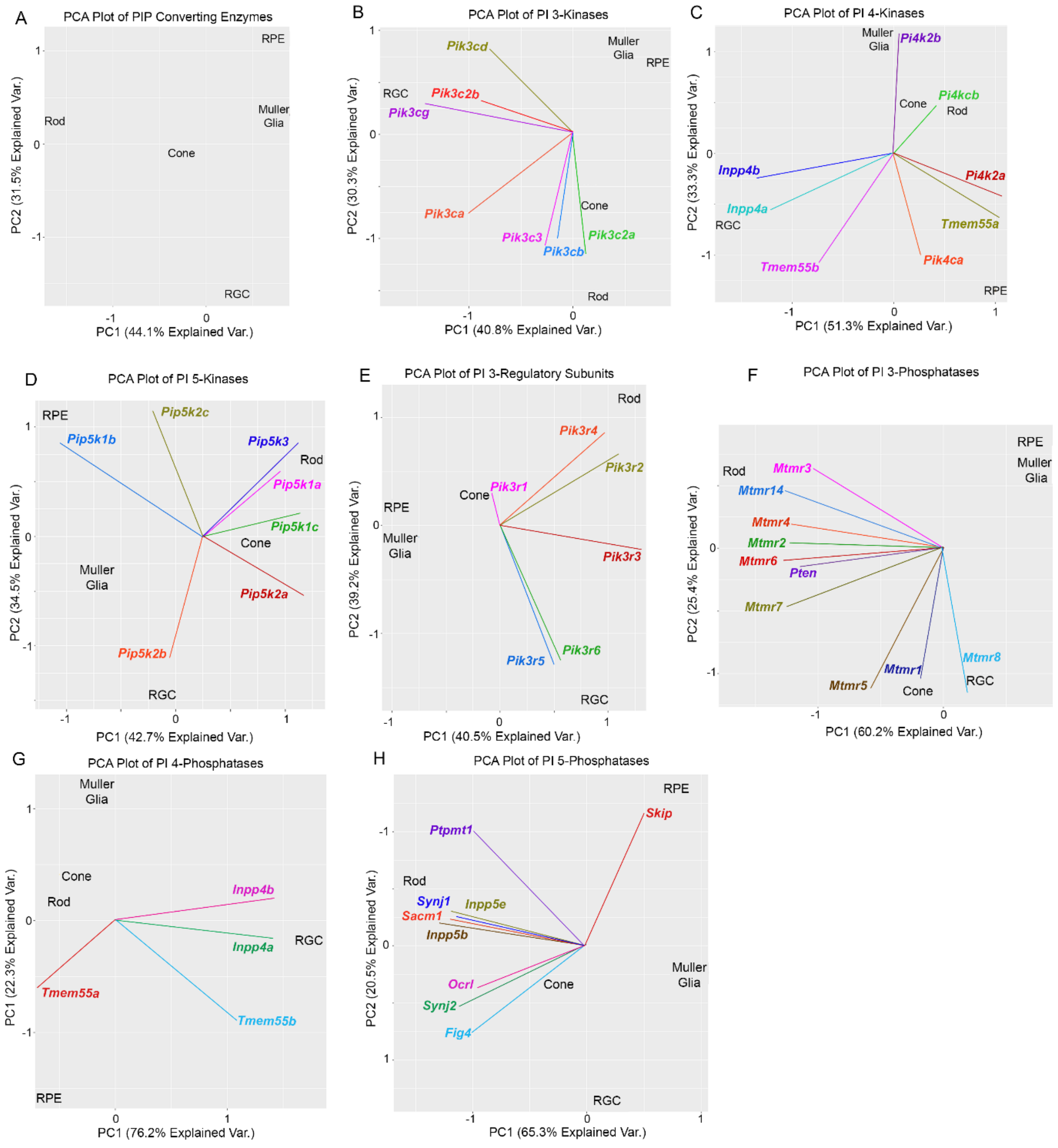


**Figure S8. Principal component analysis (PCA) of PI-converting enzymes between retinal cells.** PCA analysis between retinal cell types and the PI- converting enzymes (**A**), PI-3 Kinases (**B**), PI-4 Kinases (**C**), PI 5-kinases (**D**), PI 3-regulatory subunits (**E**), PI 3-phosphatases (**F**), PI4-phosphatases (**G**), and PI 5-phosphatases (**H**).

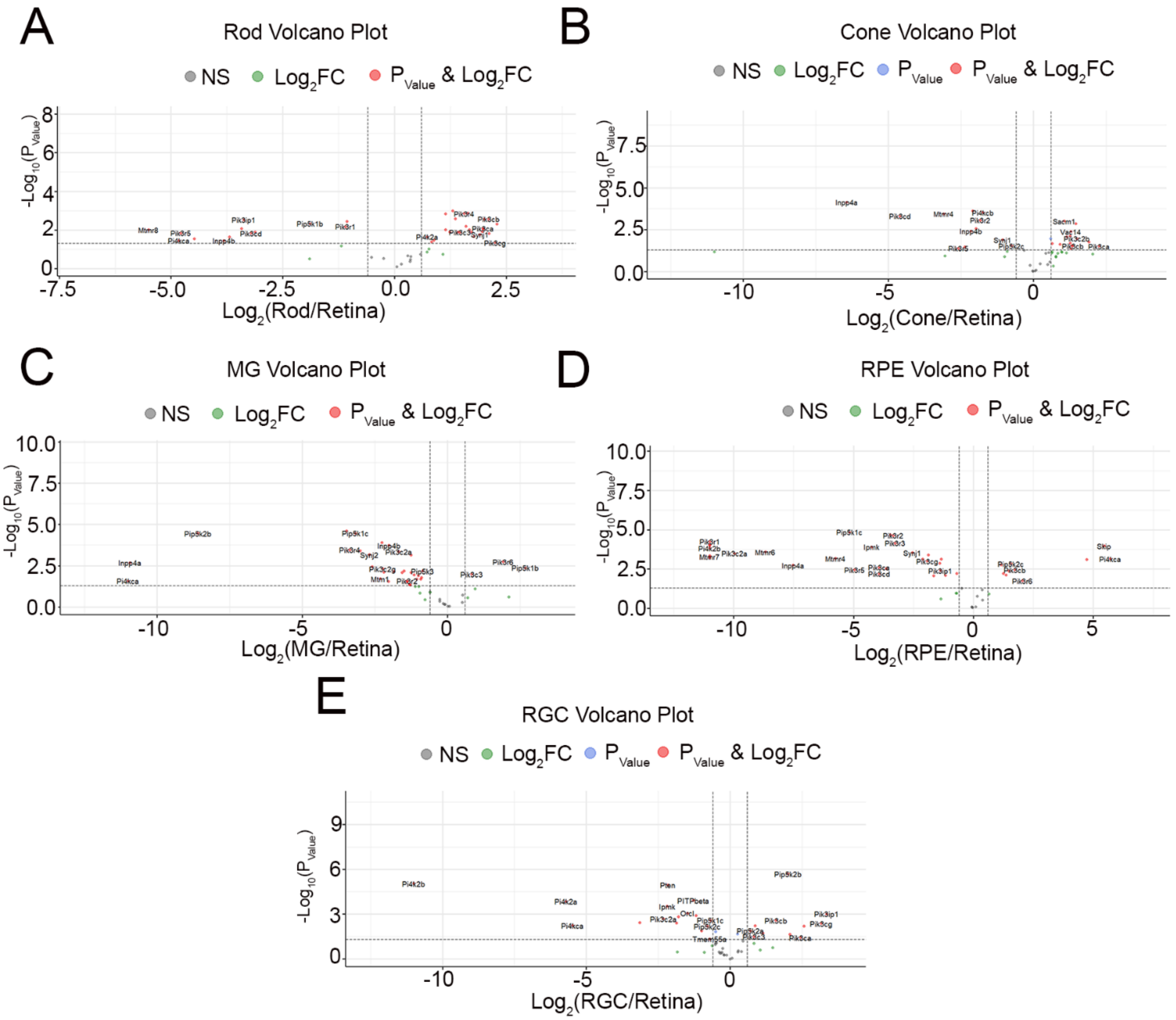


**Figure S9. The volcano plot shows the expression differential expression of PI-converting enzymes in various retinal cell types.** The volcano plot represents the combined data of both PI kinases and PI phosphatases in retina and rods (**A**); retina and cones (**B**); retina and Müller cells (**C**); retina and RPE (**D**), and retina and RGCs (**E**). The plot is divided into three main categories: bottom, no significance; left upper, significantly increased in cell types compared with the retina; the right upper significantly decreased in cell types compared with the retina. Values of no expression were rescaled to $log_2FC$=-11.

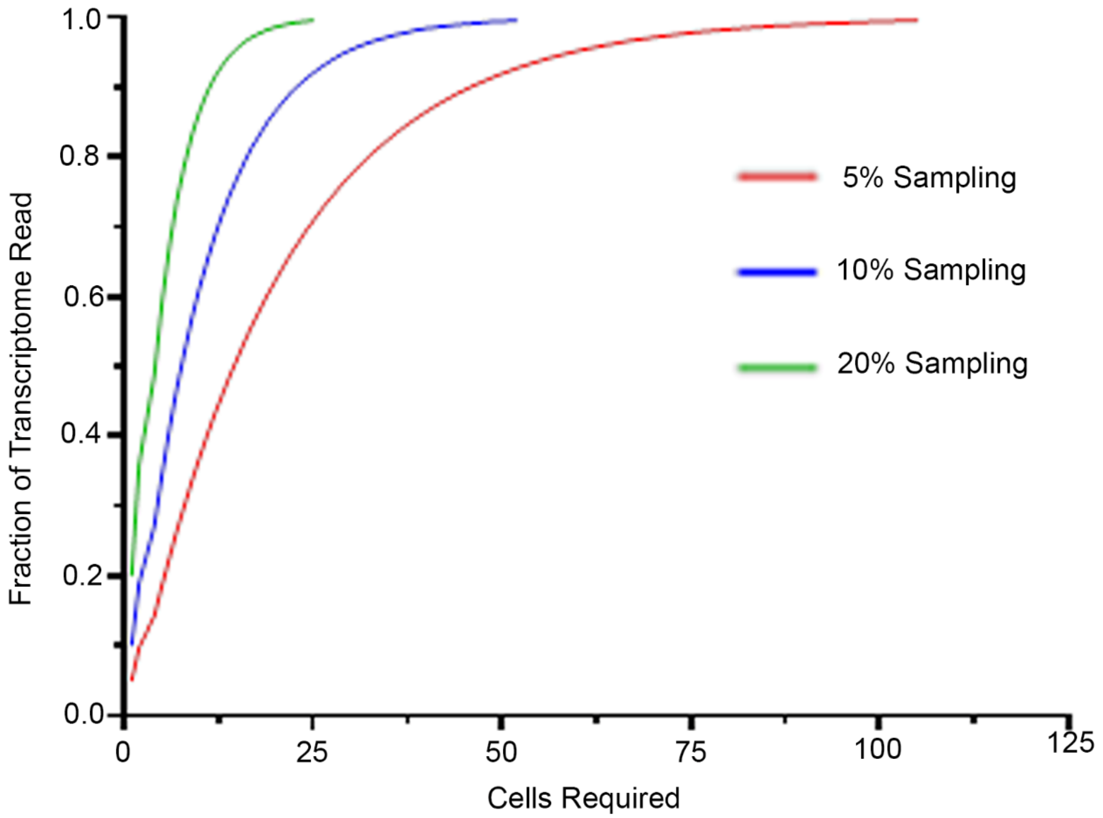


Recursive Form:
x = Sampling Rate
$N_1 = x$
$N_i = N_{i-1} + x\,(1-N_{i-1})$ for $i > 1$

Iterative Form:
x = Sampling Rate
$N_i = 1 - (1-x)^i$

i = Cell Number

**Figure S10. The sampling rate for 5-20% of reads per cell.** The number of cells required to map entire transcriptomes considering different sampling rates. We have modeled cases for 5-20% sampling rates using the equation in the figure.

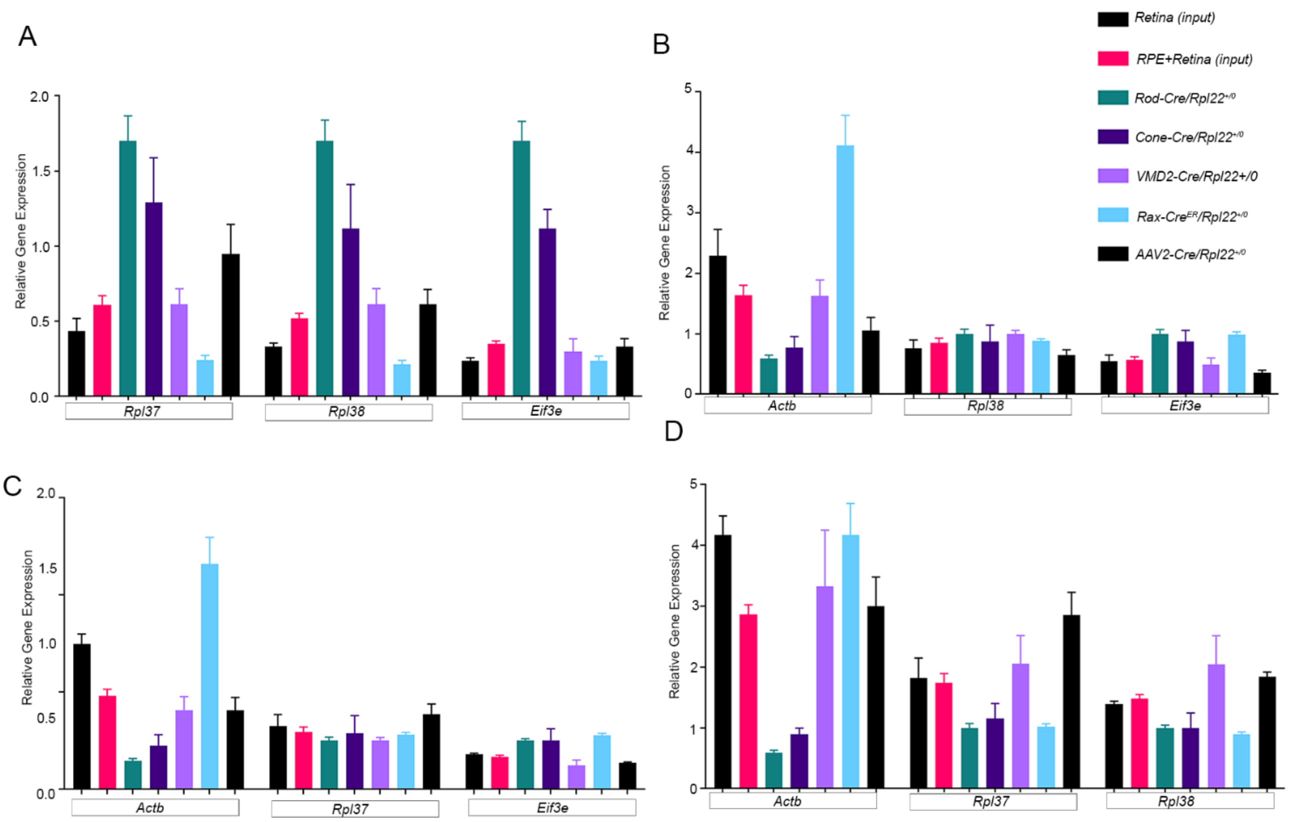


**Figure S11. Characterization of housekeeping genes.** Expression of *Actb* (Actin), *Rpl37, Rpl38* and *Eif3e* expression in RNA isolated from C57Bl6-retina (input), C57Bl6-RPE+Retina (input), *rod-Cre/Rpl22*, *cone-Cre/Rpl22*, *VMD2-Cre/Rpl22*, *Rax-Cre*$^{ER}$*/Rpl22*, and *AAV2-Cre-Rpl22* samples by qRT-PCR. *Rpl37*, *Rpl38,* and *Eif3e* levels were normalized by *Actb* (**A**); *Actb*, *Rpl38,* and *Eif3e* levels were normalized by *Rpl37* (**B**); *Atcb*, *Rpl37,* and *Eif3e* levels were normalized by *Rpl38* (**C**): and *Atcb, Rpl37,* and *RpL38* levels were normalized by *Eif3e* (**D**). Data are mean ± *SEM* (*n* = 3).

| Table S1: Retina cell type-specific primers for qRT-PCR | | | | |
|---|---|---|---|---|
| Cell type | Protein | Gene name | Forward primer | Reverse primer |
| Photoreceptor cells | Rhodopsin | *Rho* | CAAGAATCCACTGGGAGATGA | GTGTGTGGGGACAGGAGAACT |
| | Rod cGMP-specific 3',5'-cyclic phosphodiesterase subunit alpha | *Pde6a* | TCCTTGGGAGCAGCTAAAGG | CCTTCCCCCGGTAGTGAAAG |
| Cone photoreceptor cells | Short-wave length cone opsin | *Opn1sw* | TTTGGTCGCCATGTTTGTGC | AAAAGGGTGGGATGGACACC |
| Retinal pigment epithelium | RPE65 | *Rpe65* | GTTCCCCTGCAGTGATCGTT | GCAACATGAAGCCAAACCCC |
| | Lecithin retinol acyltransferase | *Lrat* | GCAGATATGGCTCTCGGATCAG | GATGCTAATCCCAAGACAGCCG |
| | Bestrophin 1 | *Best1* | CATCAGCACCTCGGTCTACAAG | GACAAGTTGGCAAACCACACCC |
| Retinal ganglion cells | Gamma synuclein | *Sncg* | CCACAAGTCCACACACGCTA | ACAGCAGCATCTGATTGGTGA |
| Bipolar cells | Transmembrane protein 215 | *Tmem215* | GGCAGGAGCCTTCAGGTAAC | ATGTCATCAGGCCGCATCTT |
| Amacrine cells | Glutamate decarboxylase 1 | *Gad1* | CCGGATCTCTCCCTTCTTCAG | GTGGTCTTGGGGTCTCTACG |
| Horizontal cells | One Cut Homeobox 1 | *Onecut1* | GCAACGTGAGCGGTAGTTTC | CAAAGCCATTTGGGGTGAGC |
| Müller glia | Glial Fibrillary Acidic Protein | *Gfap* | CAGCCTCAGGTTGGTTTCAT | CTCTCCTGTGCTGGCTACTGT |
| | Glutamine synthetase | *Gs* | GGCATAGCTGTCACAAAGCG | GTAGGGCCATCCATCAGGTG |
| | Clusterin | *Clu* | GGTGCATTCTCCGGCATTC | AGCGCTCTGCTCAAGTACAC |
| Microglia and astrocytes | Complement C1q A | *C1qa* | CAAGGGGCTCTTTCAGGTGT | GTAAATGCGACCCTTTGCGG |
| Vascular cells | Regulator of G-protein signaling 5 | *Rgs5* | TCAAAATGGCGGAGAAGGCA | GACGGTTCCACCAGGTTCTT |
| Cytoskeletal protein | Actin | *Actb* | ACTGGGACGACATGGAGAAG | GGGGTGTTGAAGGTCTCAAA |
| Ribosomal protein | Ribosomal protein L37 | *Rpl37* | CGGGACTGGTCGGATGAG | TCACGGAATCCATGTCTGAATC |
| Ribosomal protein | Ribosomal protein L38 | *Rpl38* | CGCCATGCCTCGGAAA | CCGCCGGGCTGTCAG |
| Ribosomal protein | Eukaryotic translation initiation factor 3 subunit E | *Eif3e* | GGTTGGATGCCAAGATTGATTC | GGGCGAGACTGCATTGTTG |

**Table S2: Primers for phosphoinositide kinases for qRT-PCR**

| Phosphoinositide 3-kinases | Protein | Gene name | Forward primer | Reverse primer |
|---|---|---|---|---|
| | *p110α* | *Pik3ca* | CACGATGTGAGCGGAAAGAG | AGATGGTCGTGGAGGCATTG |
| | *p110β* | *Pik3cb* | GGCATGCGGGTGTCGGA | ATAAATCCCGGTGGGCAGAAG |
| | *p110δ* | *Pik3cd* | GGGCCGAAAAGTGAATGCTG | AGCAAATAGAGCATCTGGGACAG |
| | *P110γ* | *Pik3cg* | TGGATCCATTGCCGGTTCAA | GGTGGGCAGTACGAACTCAA |
| | PI3K-C2α | *Pik3c2a* | CAGCGTGAGGTCCTGGTATT | CGAAGGGCTCAGAACAGGAG |
| | PI3K-C2β | *Pik3c2b* | CGCGCTATTGTCTCACCCG | TGGAAGACATGATGAGGGCG |
| | PI3K-C2γ | *Pikc2g* | GCCAGTTGATCCTGAGCCTT | CAGGTTGCTGTGTGTCTTGC |
| | Vps34 | *Pik3c3* | TACCTGAACGTGATGAGGCG | AGCGCATGACTCTCACAGAC |
| | p85α | *Pik3r1* | GGAGAGAGCAGGCAAATTAAA CA | TCCTTGGCTTTGCTCGGTT |
| | p85β | *Pik3r2* | CCCTACAGGCACTTGGTGTG | TGGGAGTATGTGGCCTGACT |
| | p85γ | *Pik3r3* | GACTTGTACTGGCCGTTGGA | AGGGGCTCAGAGAAGCCATA |
| | Vps15 | *Pik3r4* | ATCGCCAGCTTGTTCAGACA | CAGTCATCCCCTGTGAGAGC |
| | p101-PI3K | *Pik3r5* | CTCACCCCAACTGCTGAGAGTC | CAGTGGAACTTCGGTGGCTC |
| | p87-PI3K-adapter | *Pik3r6* | GACAGTGGAATTGAGCGGGA | GCCCTAGCATCCTGTCATCC |
| | PI3K-interacting protein 1 (inhibitor) | *Pi3kip1* | CTGAAAAACACCTCGGCTGC | CATCCTCGTCTCTTCGGCTC |
| Phosphoinositide 4-kinases | PI4K IIα | *Pi4k2a* | GCCCCATCTTGACAATCCCA | TCCCCCAAAGGAAACTGGAC |
| | PI4K IIβ | *Pi4k2b/ Pi4ka* | CCGCACTACGAGCTCAGAAA | ACTTCCACTTGACCCTTGAGA |
| | PI4K IIIα | *Pi4kca/ Pi4kb* | CACCTCCTGTCTCAGGTTCAA | TTACCTCTGCCTTTCCGAGC |
| | PI4K IIIβ | *Pi4kcb* | GGCAACCGGCTCTTCTACTT | CGGACAGGGGAACTGAATGAA |
| Phosphoinositide 5-kinases | PIPK 1α | *Pip5k1a* | CGCAATACCGGGGTTTCCTT | CCGCGTCTCGGATAGAACAA |
| | PIPK Iβ (PIP5K1B) | *Pip5k1b* | GAGAACCCACGACATCCCGA | CAGGTACGGCGTCTCCATTT |
| | PIPK Iγ (PIP5K1C) | *Pip5k1c* | CACGGCCATGGAGTCTATCC | GAACTCTTCCGGAACACCGT |
| | PIPK IIα (PIP5K2A) | *Pip4k2a/ Pip5k2a* | GCCACGTTCAAATCCCTGTC | GGGGTGCACTTCTGGTCAAG |
| | PIPK IIβ (PIP5K2B) | *Pip4k2b/ Pip5k2b* | GCATGTCGTCCAACTGCACC | GGCCCGGAATAGCTTCACTT |
| | PIPK IIγ (PIP5K2C) | *Pip4k2c/ Pip5k2c* | AGGACCTAAGCCTAAGCGGA | GAACAGTCGGGAACAGTCGT |
| | PIPK III (PIKFyve or PIP5K3) | *Pip5k3/ Pikfyve* | GATTCATCCGGATTCCTCAA | TAGCCTGGGGACTGACAGAT |
| Other | Inositol-polyphosph ate multikinase (IPMK) | *Ipmk* | TGAAGATTGGGCGGAAGAGC | GCCATTGTGGAAAAACTTGG |

| Table S3: Primers for phosphoinositide phosphatases for qRT-PCR | | | | |
|---|---|---|---|---|
| | Protein | Gene name | Forward primer | Reverse primer |
| Phosphoinositide 3-phosphatases | PTEN | *Pten* | GGAGCAAGGCTTGTAGTGGT | CCATTGGTAGCCAAACGGAAC |
| | MTM1 | *Mtm1* | GAACTTACTGGCTGGTCAGG | GTGCCAGAGGAAAGGCATGT |
| | MTMR1 | *Mtmr1* | CCTCCAGTTTCTCGCGCCAT | CCCGTGACCTAAAGGATGCC |
| | MTMR2 | *Mtmr2* | GCTGGGAGCAGGTGGATAAA | CCCACAATTCCTGCAGTGGT |
| | MTMR3 | *Mtmr3* | CGCCAAGGTAGAATGGGTGA | CTTGAGAGCCACTCTTGGCA |
| | MTMR4 | *Mtmr4* | CCACATCTAGCTCTCGGCAG | GGACAAACCAACGGGCTTTC |
| | MTMR5 | *Mtmr5/ Sbf1* | CCACATCTAGCTCTCGGCAG | GGACAAACCAACGGGCTTTC |
| | MTMR6 | *Mtmr6* | GGACAACCAAGGTTGAACAAGT | ATTGCATTGAGCTTGGGCCT |
| | MTMR7 | *Mtmr7* | GAAAACGTGCGCTTGGTAGA | CGTGCAAGGCGTATCAGAGA |
| | MTMR8 | *Mtmr8/ Mtmr9* | CCGTCAGACGCCGGT | GTACATCAGGGTGACCGAGT |
| | MTMR14 | *Mtmr14* | AGGAGTTCTCCCGGACTCAG | CGGCCAAATAGCTCCAGACA |
| Phosphoinositide 4-phosphatases | INPP4A | *Inpp4a* | CCACGTGGTCCAAAAGCAAG | TTATGTTGCCGACACGGTCA |
| | INPP4B | *Inpp4b* | CACCGTGGAGAATAGGTCCG | TGGAGTACCTCGTCAGGGTC |
| | TMEM55A | *Tmem55a* | CGTACTTGCAGGAAAGCAGC | GGACATCCTATTCGCCGAGA |
| | TMEM55B | *Tmem55b* | ACGGAGCCGGTAAACATGC | CACAGATGACCCTGACACCC |
| Phosphoinositide 5-phosphatases | SYNJ1 | *Synj1* | GGACGCTTCCTGAGCGGT | CCCCACACATGAGCCGTAAT |
| | SYNJ2 | *Synj2* | CTGTGGGCCGAGCTATTGTC | CCCATTCAGAGCCGTCATCA |
| | OCRL1 | *Ocrl* | ATTGGAGGCTTTGTGCCGAA | TAGGTAGCTGTCTTCTTCCAGGT |
| | INPP5B | *Inpp5b* | CTGAGACCGTAGGGACAGGA | CACAGGATTCGGTCACACCA |
| | INPP5E | *Inpp5e* | AGGGGCATCCACTCTAGTCT | GGCAGGATTATGAAGTCCAGGG |
| | SKIP | *Skip/ Inpp5k* | ATAAGCCTGTCACTGGCACC | GACGCATCCCCACCTTGTAT |
| Other | PLIP | *Ptpmt1* | GAAGCGATCGCCAAAATCCG | GGTCGGGTTAAGCTGCTTTG |
| | SAC1 | *Sacm1l* | AGAGGTCACCCTTGCAGTCA | ACATCAAAATCTGTGGCTCTCCA |
| | SAC3 | *Fig4* | GCTGGTTCATCGGGTAAAGA | GGCTCATGGTGTTTTTGTGA |
| | PITPβ | *Pitpnb/ Pitpβ* | TCAGGTTGGACAGCTTTACTCT | GTGTACTGTCCCTTCTCGCC |
| | VAC14 | *Vac14* | CTGCTGGACGTGAAGAACAAC | CTAGGGCCCTTTTCCATGCT |

| **Table S4: Opti-prep Samples Compared to Photoreceptor (Enriched Genes) [Figure S7D]** | | |
|---|---|---|
| Groups | Number of Genes | Gene Name |
| Rod Cone | 7 | *Sacm1 Skip Pik3cb Pik3ca Ipmk Pip5k2a Vac14* |
| Optiprep Rod | 4 | *Pik3c3 Ptpmt1 Mtmr3 Mtmr4* |
| Cone Optiprep | 1 | *Pik3c2b* |
| Rod | 14 | *Mtmr6 Mtmr2 Inpp5b Tmem55a Pip5k3 Pik3r4 Pik3cg Pi4k2a Synj1 Pik3r2 Mtmr14 Mtmr7 Pi4kcb Inpp5e* |
| Cone | 6 | *Pik3ip1 Pik3r1 Pi4k2b Pik3c2a Mtmr8 Pip5k1b* |
| Optiprep | 6 | *Pip5k2b Mtm1 Pip5k2c Synj2 Pik3r5 Pik3r6* |

| **Table S5: Opti-prep Samples Compared to Photoreceptor (Depleted Genes) [Figure S7E]** | | |
|---|---|---|
| Groups | Number of Genes | Gene Name |
| Rod Cone | 4 | *Pik3cd Pik3r5 Inpp4b Inpp4a* |
| Optiprep Rod | 3 | *Pik3r1 Pi4kca Pik3c2g* |
| Cone Optiprep | 1 | *Tmem55b* |
| Rod | 6 | *Pik3ip1 Pip5k2b Mtm1 Pip5k1b Pik3r6 Mtmr8* |
| Cone | 7 | *Synj1 Pik3r2 Pip5k2c Pip5k1c Synj2 Mtmr4 Pi4kcb* |
| Optiprep | 6 | *Ocrl Vac14 Mtmr6 Pi4k2b Pik3c2a Pip5k3* |

| **Table S6: Enriched PI Converting Genes Among Retinal Cells [Figure 4A]** | | |
|---|---|---|
| Groups | Number of Genes | Gene Name |
| Cone RGC RPE Rod | 1 | *Pik3cb* |
| Cone RPE Rod | 1 | *Skip* |
| Cone RGC Rod | 3 | *Pik3ca* *Pip5k2a* *Vac14* |
| MG RGC Rod | 1 | *Pik3c3* |
| Cone RGC RPE | 1 | *Pik3c2b* |
| MG RGC RPE | 1 | *Pik3r6* |
| RGC Rod | 1 | *Pik3cg* |
| Cone Rod | 2 | *Sacm1* *Ipmk* |
| Cone RGC | 2 | *Pik3ip1* *Mtmr8* |
| RPE Rod | 3 | *Ptpmt1* *Tmem55a* *Pi4k2a* |
| Rod | 13 | *Mtmr6* *Mtmr2* *Inpp5b* *Pip5k3* *Pik3r4* *Mtmr3* *Synj1* *Pik3r2* *Mtmr14* *Mtmr7* *Mtmr4* *Pi4kcb* *Inpp5e* |
| Cone | 3 | *Pik3r1* *Pi4k2b* *Pik3c2a* |
| RPE | 2 | *Pip5k2c* *Pi4kca* |
| RGC | 1 | *Pip5k2b* |

| **Table S7: Depleted PI Converting Genes Among Retinal Cells [Figure 4B]** | | |
|---|---|---|
| Groups | Number of Genes | Gene Name |
| Cone MG RPE Rod | 3 | *Inpp4b Inpp4a Pik3r5* |
| Cone MG RGC RPE | 2 | *Pip5k1c Pi4kcb* |
| Cone RPE Rod | 1 | *Pik3cd* |
| MG RPE Rod | 2 | *Pip5k2b Pik3c2g* |
| MG RGC Rod | 1 | *Pi4kca* |
| Cone MG RPE | 4 | *Synj2 Synj1 Pik3r2 Mtmr4* |
| Cone MG RGC | 1 | *Pip5k2c* |
| MG RGC RPE | 4 | *Pten Pik3c2a Inpp5b PITPbeta* |
| MG Rod | 1 | *Mtm1* |
| RPE Rod | 2 | *Pik3ip1 Pik3r1* |
| Cone MG | 1 | *Tmem55b* |
| MG RPE | 7 | *Mtmr6 Mtmr2 Pip5k2a Pik3r3 Vac14 Mtmr5 Mtmr7* |
| MG RGC | 4 | *Ocrl Sacm1 Pip5k3 Mtmr14* |
| RGC RPE | 2 | *Pi4k2b Ipmk* |
| Rod | 3 | *Mtmr8 Pip5k1b Pik3r6* |
| MG | 1 | *Pik3r4* |
| RPE | 3 | *Pik3ca Pik3cg Fig4* |
| RGC | 2 | *Tmem55a Pi4k2a* |

| **Table S8: PI Converting Genes Among Photoreceptors [Figure 4C]** | | |
|---|---|---|
| Groups | Number of Genes | Gene Name |
| Rod-Cone Enriched (PR Specific) | 7 | *Sacm1 Skip Pik3cb Pik3ca Ipmk Pip5k2a Vac14* |
| Rod-Cone Depleted (PR Non-Specific) | 4 | *Inpp4b Inpp4a Pik3cd Pik3r5* |
| Rod Enriched-Cone Depleted (Rod Specific) | 4 | *Synj1 Pik3r2 Mtmr4 Pi4kcb* |
| Cone Enriched-Rod Depleted (Cone Specific) | 4 | *Pik3ip1 Pik3r1 Mtmr8 Pip5k1b* |
| Rod Enriched | 14 | *Mtmr6 Pik3c3 Mtmr2 Ptpmt1 Inpp5b Tmem55a Pip5k3 Pik3r4 Pik3cg Mtmr3 Pi4k2a Mtmr14 Mtmr7 Inpp5e* |
| Cone Enriched | 3 | *Pi4k2b Pik3c2a Pik3c2b* |
| Rod Depleted | 5 | *Pip5k2b Mtm1 Pik3c2g Pi4kca Pik3r6* |
| Cone Depleted | 4 | *Pip5k2c Synj2 Tmem55b Pip5k1c* |
